# A Seascape Genomics Perspective on Restrictive Genetic Connectivity Overcoming Signals of Local Adaptations in the Green Abalone (*Haliotis fulgens*) of the California Current System

**DOI:** 10.1002/ece3.70913

**Published:** 2025-02-04

**Authors:** Jorge Alberto Mares‐Mayagoitia, Paulina Mejía‐Ruíz, Fabiola Lafarga‐De la Cruz, Fiorenza Micheli, Pedro Cruz‐Hernández, Juan A. De‐Anda‐Montañez, John Hyde, Norma Y. Hernández‐Saavedra, Vladimir S. De Jesús‐Bonilla, Carmen E. Vargas‐Peralta, Ana L. Flores‐Morales, Alejandro F. Pares‐Sierra, Fausto Valenzuela‐Quiñonez

**Affiliations:** ^1^ Programa de Ecología Pesquera Centro de Investigaciones Biológicas del Noroeste S.C. La Paz Baja California Sur Mexico; ^2^ Departamento de Acuícultura‐Departamento de Oceanografía Física Centro de Investigaciones Científicas y de Educación Superior de Ensenada Ensenada Mexico; ^3^ Oceans Department and Stanford Center for Ocean Solutions, Hopkins Marine Station Stanford University Pacific Grove California USA; ^4^ NOAA Fisheries Southwest Fisheries Science Center La Jolla California USA; ^5^ Facultad de Ciencias Marinas Universidad Autónoma de Baja California Ensenada Mexico

**Keywords:** genetic connectivity, *Haliotis fulgens*, marine conservation, northeast pacific, population genomics, seascape genomics

## Abstract

Seascape genomics facilitates integrative research on eco‐evolutionary forces, such as migration and natural selection, which shape genomic connectivity and structure and provide critical insights for conservation strategies. The green abalone ( *Haliotis fulgens* ) is distributed from California, United States, to Baja California Sur, Mexico, and exposed to a latitudinal environmental gradient in the California Current System. This study aimed to investigate genomic population structure and potential local adaptations of green abalone across its distribution. The green abalone exhibits a distinctive neutral genetic structuring influenced by geographic distance and marine currents rather than local adaptations. Analyses using 9100 neutral and 17 outlier SNPs revealed three distinct populations: the North group (California to Ensenada, Baja California), a population on Guadalupe Island, and the South group (coastal locations of the Baja California peninsula). The research underscores the significance of life history traits and larval dispersal in shaping genetic connectivity. Connectivity appears to be influenced by geographic distance on neutral genetic structure, overshadowing natural selection's role. Furthermore, no genome–environment associations to sea surface temperature values were found. Future research should integrate genetic data with ocean circulation modeling to better understand the mechanisms and outcomes of larval dispersal and genetic connectivity. This study emphasizes the importance of both local and binational (USA‐Mexico) conservation efforts, suggesting the development of SNP marker panels for traceability and management. Collaborative strategies could serve as models for binational conservation initiatives in other ecoregions, promoting sustainable management and conservation of green abalone populations and other exploited species across national borders.

## Graphical abstract

The green abalone exhibits a distinctive neutral genetic structuring influenced by geographic distance and marine currents rather than local adaptations. Analyses using 1900 neutral and 17 putative outlier SNPs revealed three distinct populations: the North group (California to Ensenada, Baja California), a population on Guadalupe Island, and the South group (coastal locations of the Baja California peninsula). Connectivity appears to be influenced by geographic distance on neutral genetic structure, overshadowing natural selection role.

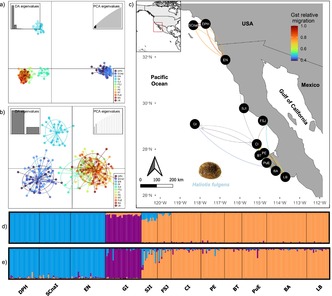

## 1 Introduction

Migration and natural selection are critical eco‐evolutionary forces influencing population structure (Selkoe et al. 2016). Gene flow, which involves the exchange of genetic material among populations, is introduced by migration and tends to homogenize genetic differences and promote genetic cohesion (Slatkin 1987). Conversely, local adaptations arise from specific selective pressures in distinct environments, leading to genetic differentiation (Kawecki and Ebert 2004). The balance between these processes creates complex population structures where geographical regions might exhibit high genetic similarities due to gene flow, and others show significant genetic divergence driven by local adaptation pressures (Liggins, Treml, and Riginos 2020).

Local adaptations can persist and even spread in populations with high levels of gene flow (Yeaman and Whitlock 2011). Despite the homogenizing effect of gene flow, which reduces genetic differences, strong local selective pressures can develop and maintain adaptive traits (Lenormand 2002). This indicates that local adaptations are not necessarily overwhelmed by gene flow; instead, they can coexist and evolve within highly connected populations, demonstrating the power of natural selection in maintaining adaptive divergence, which commonly results in different spatial patterns between neutral and outlier loci (Sexton, Hangartner, and Hoffmann 2014; Selmoni et al. 2020; Segovia, González‐Wevar, and Haye 2020). However, ocean currents, geographic barriers, and other physical factors may limit the dispersal of individuals, leading to isolated populations with reduced gene flow (White et al. 2010) and broadly similar patterns between neutral and adaptive genomic structuring, which have been recently observed in different marine species, such as in the common cockle ( *Cerastoderma edule* ) (Coscia et al. 2020), the giant black tiger shrimp ( *Penaeus monodon* ) (Vu et al. 2021), and the blenny ( *Eleginops maclovinus* ) (Canales‐Aguirre et al. 2022).

The distribution of green abalone ( *Haliotis fulgens* ) ranges from Point Conception, California, USA, to Bahia Magdalena, Baja California Sur, Mexico (Guzmán‐del Próo 1992). This is a heterogeneous area influenced by the California Current System (CCS), driven year‐round mainly by the California Current southwards the Pacific coasts of California and the Baja California Peninsula (BCP) (Lynn and Simpson 1987; Auad, Roemmich, and Gilson 2011) and transporting temperate, cold nutrient‐rich waters, exerting a significant influence in summer with persistent eddies (Chenillat et al. 2018). On the contrary, a warmer countercurrent is found near the coasts, intensively in the autumn and winter seasons along the south‐central region of the BCP, around Punta Eugenia (Durazo 2015). Also, this region is described to belong to the warm‐temperate Northeast Pacific biogeographic province, which includes the Southern California Bight (SCB), from Point Conception, California, to approximately Punta Eugenia in BCS, and the Magdalena Transition (MT), from Punta Eugenia to the end of BCS marine ecoregions (Spalding et al. 2007).

The green abalone is a broadcast spawner, reaching sexual maturity at a shell length of 6–12 cm (Sierra‐Rodríguez et al. 2006), and features peak reproductive behavior in autumn–winter and winter–spring seasons (Vélez‐Arellano et al. 2020). Larval development is considered temperature‐dependent, and the swimming larval phase is estimated to last from 4 to 15 days with an optimal temperature range from 20°C to 23°C (Leighton 2000), settling and developing in rocky patches from the intertidal zone to ~24 m in depth (Sierra‐Rodríguez et al. 2006).

The status of green abalone is under protection in California USA due to depleted populations (CDFG 2005) and setbacks regarding the Alle effect, which can lead to low external fertilization success in marine spawners with sedentary or sessile adult stages, and in reproductive and recruitment efforts (Allee et al. 1949). It is also under strict management strategies in Mexico, including active participation of the Federation of Cooperative Societies of Baja California (FEDECOOP) (McCay et al. 2014). These contrasting transboundary management strategies reinforce the necessity of determining its evolutionary units and developing tools for accurate population assignment along its distribution range.

Most of the earlier studies on genetic‐population structure in abalone species in the region have focused on geographically restricted areas and/or limited number of neutral markers (Hamm and Burton 2000; Gutiérrez‐González et al. 2007; Díaz‐Viloria et al. 2009; Gruenthal et al. 2014; Munguía‐Vega et al. 2015; Mejía‐Ruiz et al. 2020; Mares‐Mayagoitia et al. 2021), which makes it difficult to accurate determine the population structure, genetic connectivity and potential local adaptation along the entire distribution range, and assess the possible impacts on binational management schemes.

Current evidence on the neutral genomic structure of green abalone in part of the Mexican Pacific highlighted the existence of three populations integrated for (1) Guadalupe Island, (2) southern coastal locations south of Punta Eugenia, and subtle (3) northern coastal locations (Mejía‐Ruíz et al. 2020). Furthermore, moderate to high population assignment (> 75%) has been achieved in the region (Mejía‐Ruíz et al. 2023). However, the extent of the northern coastal population and the ecological drivers that shape evolutionary units remain unknown, reinforcing the need for a large‐scale analysis of its distribution range.

Therefore, the green abalone is an excellent study model to test the marked genomic‐neutral structure of the green abalone in systems with contrasting environments where selection may act, as previously reported on several species (Stanley et al. 2018). We hypothesized that the adaptive divergence of green abalone would mirror environmental patterns in the area; as for neutral population structure, it would be more complex and mainly driven by isolation by distance effect (IBD). The main objective of this research is to contrast the underlying causal drivers of the genomic population structure and local adaptation of the green abalone.

## 2 Material and Methods

### 2.1 Sample Collection

A total of 303 adult green abalone were sampled from 12 locations across their distribution range in southern California, USA, and the Baja California Peninsula, Mexico. Samples were collected by SCUBA or hooka divers from depths of 5 to 15 m. NOAA Southwest Fisheries Science Center provided California samples. Baja California Peninsula samples (EN, GI, SJI, FSJ, CI, PE, BT, PuE, BA, and LB) were collected by Centro de Investigaciones Biológicas del Noroeste researchers and commercial fishery cooperatives. The abalone tissue was preserved in 96% ethanol.

### 2.2 Genomic Libraries Preparation and Sequencing

First, genomic DNA extraction was conducted using the chloroform: isoamyl alcohol protocol (Sambrook and Russell 2001) with an added RNase treatment (New England Biolabs, Ipswich, MA, USA). Genomic library preparation consisted of ddRAD‐Seq protocol (Peterson et al. 2012) and the next specifications: a double‐digestion with two restriction enzymes, EcoRI‐HF (NEB) and MspI (NEB), followed by the ligation of specific adapters (P1–P2) with T4 ligase (New England Biolabs, Ipswich, MA, USA) to adhere to the cohesive ends, and adding an index to all 48 different barcodes and mixed in equimolar concentrations to create a pool. DNA fragment size was selected (376 ± 50 bp) for each pool using Pippin Prep (Sage Science, Beverly, MA, USA). California libraries were processed at the Genetic Aquaculture lab in CICESE and sequenced on an Illumina Hi‐Seq X with 150 bp paired‐end at the Novogene facility (Sacramento, USA). While Baja California Peninsula libraries were constructed at the Centro de Investigaciones Biológicas del Noroeste and sequenced on two lanes of an Illumina Hi‐seq 4000 platform at Novogene (Sacramento, CA, USA), using 150‐bp pair‐end reads.

### 2.3 Reference Genome Alignment, SNP Filtering, and Datasets

Raw sequences were demultiplexed using Stacks 2.54 (Rochette, Rivera‐Colón, and Catchen 2019) setting the next parameters in *process_radtags*: rescue barcodes and RAD‐Tag cut sites (‐r), remove any reads with an uncalled base (‐c) and reads with low‐quality scores (‐q) using a minimum Phred score of 25 (‐s).

Demultiplexed reads were mapped to the green abalone ( *Haliotis fulgens* ) reference genome (downloaded from the Abalone Genomics Resources Website of Iowa State University; http://abalone.dbgenome.org/downloads) using the BWA‐MEM algorithm in bwa 0.7.12 (Li and Durbin 2009). Parameters for the reference genome assembly filtering process were the grep function to retain unique alignments, quality alignments greater than MapQ 30 (‐q), keeping the properly mapped paired reads (‐f 0x02), removing supplementary (‐F 0x800) and unmapped reads (‐F 0x04), and sorting properly paired alignments using the *view* command in samtools 1.4 (Li et al. 2009).

The *ref_map.pl* pipeline in Stacks was performed for genotypes and variant sites calling at a 5% significance level in *gstacks* (–var_alpha 0.05 and –gt‐alpha 0.05). The *populations* component of Stacks filtered genotype SNPs using the next parameters: consider a locus when present in 80% of the individuals (‐*r* = 0.8) and every sampling location (‐*p* = 12), the minor allele frequency > 3% (*min_maf* = 0.03) implemented to differentiate between common and rare variants in putative populations, and the command ‐*write_single_snp* to restrict only the first SNP per *locus*.

Missing data were evaluated per individual (*–missing‐indv*) and locus (*–max‐missing*) using vcftools v.3.0 (Danecek et al. 2011), removing those loci and individuals with more than 10% and 17% of missing data, respectively. Genotypes were filtered with > 3 depth coverage with the R package SNPfilter (DeRaad 2022), and the filtered vcf and genepop files were converted when necessary for subsequent analyses using PGDSPIDER v.2.1.1.5 (Lischer and Excoffier 2012).

### 2.4 Detecting Outlier Loci

Two independent methods were required to consider loci under potential selection. First, Bayescan v.2.1 (Foll and Gaggiotti 2008) by setting a *pr_odds* of 100, a burn‐in of 50,000 steps, and 100,000 iterations, and results were corrected using a 0.05 false discovery rate (FDR) with *q*‐values logarithm. Second, PCAdapt v.4.3.3 (Luu, Bazin, and Blum 2017; Privé et al. 2020) using a principal components analysis (PCA) to determine the number of populations (*k* = 3), and outlier SNPs were detected after the distribution of *p* values was corrected by an FDR with *q*‐values of 0.05. Loci detected in common between methods were considered outliers.

### 2.5 Neutral Dataset: Hardy–Weinberg Equilibrium and Genetic Diversity

The neutral dataset was built after the Hardy–Weinberg equilibrium test (HWE) in GenoDive 3.05 (Meirmans 2020) using 10,000 iterations, and no loci in disequilibrium were identified in any of the 12 sampling locations after Bonferroni sequential correction. Subsequently, loci were filtered by pairwise linkage disequilibrium (LD) estimations among all loci using the *r* ^*2*^ (> 0.25) method in PLINK v.1.90 (Purcell et al. 2007).

The genetic diversity indexes: observed (*Ho*) and expected (*He*) heterozygosity, and inbreeding coefficient (*Fis*) were estimated for each sampling location using the ADEGENET 1.3.1 (Jombart and Ahmed 2011) and poppr (Kamvar, Tabima, and Grünwald 2014) packages in R.

### 2.6 Genomic Structure

A genetic differentiation analysis and two clustering analyses were conducted for neutral and outlier loci for a wide perspective of green abalone's genetic structure. Pairwise population differentiation was first calculated by the *FST* estimator (Weir and Cockerham 1984) in the R package StAMPP v 1.6.3 (Pembleton, Cogan, and Forster 2013), parameters were set at 10,000 permutations for each pairwise comparison, and the significance level was corrected for multiple comparisons with the sequential Bonferroni correction (Holm 1979).

Secondly, clustering analyses started with a discriminant analysis of principal components (DAPCs) (Jombart, Devillard, and Balloux 2010) in the R package ADEGENET v 1.3.1 (Jombart and Ahmed 2011), a non‐model‐based method for maximizing differences among groups and minimizing variation within them. The xvalDapc function was used to obtain the optimal number of PCs retained for the optimal number of clusters assessment with the *find.clusters* function and the Bayesian information criterion (BIC) method to avoid model overfitting.

Third, STRUCTURE v 2.3.4 (Pritchard, Falush, and Stephens 2002; Falush, Stephens, and Pritchard 2003), a Bayesian clustering algorithm was used to infer the more likely number of putative populations (*K*), testing up to 13 potential genotypic clusters among individuals (*K* = 1–13) with five replicates for each *K* value. Parameters consisted of the admixture model and correlated allele frequencies applied with a 10,000 burn‐in and 100,000 iterations of Markov chain Monte Carlo (MCMC) steps, including prior information on sampling location. Then combining STRUCTURE analysis and STRUCTURE HARVESTER (Earl and VonHoldt 2012), the StrAuto program (Chhatre and Emerson 2017) was implemented to infer the optimal *K* value using the ΔK statistic (Evanno, Regnaut, and Goudet 2005). Results of the best *K* were compiled with CLUMPP v 1.1.2 (Jakobsson and Rosenberg 2007) and then visualized with DISTRUCT v 1.1 (Rosenberg 2004) software.

### 2.7 Genetic Connectivity and IBD Analyses

Genetic connectivity and directional relative migration rates were estimated among the sampling locations using *G*_*ST*_ values (Nei 1973) for the neutral dataset with the divMigrate function (Sundqvist et al. 2016) of the R package diveRsity (Keenan et al. 2013). The parameters implemented consisted of 1000 bootstrap repetitions and an arbitrary filter threshold of 0.45. Genetic connectivity patterns were visualized using network graphics produced with the R package qgraph (Epskamp et al. 2012).

Then, IBD was estimated by comparing the matrices of standardized genetic distance (*F*_*ST*_/(1‐*F*_*ST*_)) of neutral loci and geographic distance. Geographic distances were calculated in kilometers with the shortest water path among locations using Google EarthTM and the function *mantel.randtest* was implemented to run a mantel test in the R package ADEGENET v 1.3.1 (Jombart and Ahmed 2011) using 9999 iterations. Two IBD tests were performed, one considering all 12 locations and the other hierarchical IBD with only BCP locations (except for EN location).

### 2.8 Perspective on Genomic–Environment Associations

**FIGURE 1:**
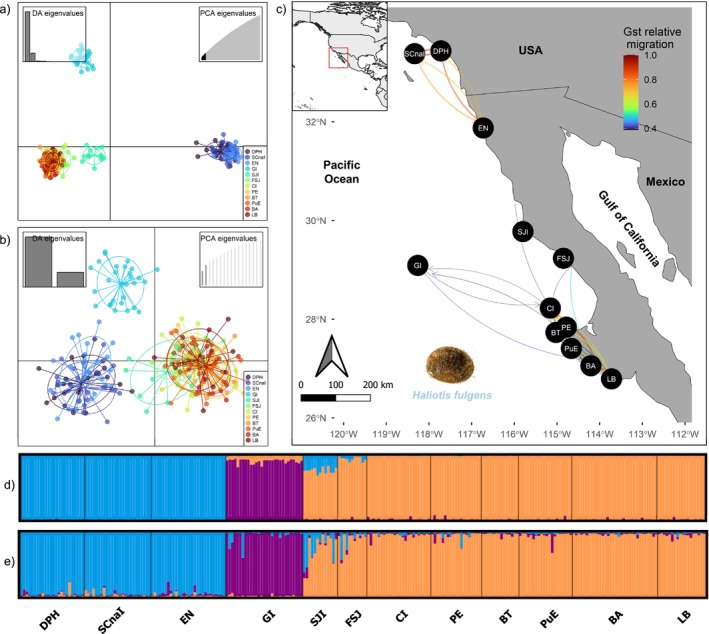
Study area and genomic structure: Discriminant analysis of principal components (DAPCs) for green abalone ( *Haliotis fulgens* ) for 9100 neutral loci (a); DAPC for 17 outlier loci (b). Relative migration networks based on the neutral SNPs among 12 sample locations using the Nei's Gst estimated in divMigrate, only migration rates > 0.45 are shown and circles represent localities, while arrows specify the direction and magnitude of relative migration levels. The left and right arrows indicate a southward or a northward direction respectively (c). Individual population assignment values based on the *K* method (*K* = 3) for (d) 9100 neutral loci and (e) 17 outlier loci. Sampling locations: Dana Point Harbor (DPH), Santa Catalina Island (SCnaI), Ensenada (EN), Guadalupe Island (GI), San Jerónimo Island (SJI), Faro San José (FSJ), Cedros Island (CI), Punta Eugenia (PE), Bahía Tortugas (BT), Puerto Escondido (PuE), Bahía Asunción (BA), and La Bocana (LB).

Polygons of fisheries concession areas in BCP and arbitrary surrounding areas for each sampling location in California (Figure 1c) were used to extract environmental data from two publicly online resources: The AquaModis satellite (https://coastwatch.pfeg.noaa.gov/erddap/griddap/) for sea surface temperature and chlorophyll variables, and the EU Copernicus Marine Service (2017) for pH and dissolved oxygen variables. The extracted environmental datasets compiled records for the 2002–2019 period, processing monthly mean, maximum, and minimum values in R using the *raster* package v.2.8 (Hijmans et al. 2015).

### 2.9 Seascape Variable Structure and GEA's Marker Detection

The environmental structure of the sampling polygons belonging to the California currents system was characterized through an environmental principal components analysis (ePCA) using the *prcomp* function in the *stats* package from the R environment (R Development Core Team 2008). The 12 environmental variables used were standardized with the *scale* function, and the first two PCs were retained.

The selection of environmental variables to build the environmental matrix for the genotype–environment association analyses (GEA) was first through the variable that contributed the most to each of the three retained components, identified with the *which.max* function applied to the loadings, and secondly by considering the Spearman's correlation coefficients among all environmental variables, calculated to evaluate collinearity from the multiple logistic regression (Table S11). The most biologically relevant variables were considered to build the environmental matrix for the GEA analysis.

To detect genome–environment association markers, we performed two analyses: The latent factor of mixed models v 1.4 (LFMM, Frichot et al. 2013), and redundancy analysis (RDA). The LFMM correlates environmental variables and genotypic variation, considering the population structure (K) and the environmental variables as latent factors. The parameters used consisted of 100,000 iterations after a burn‐in of 10,000 first steps and five independent runs for each analysis performed in the R LEA package (Frichot and Francois 2015). The significance values of the *z*‐scores associated with each SNP were calculated using *α* = 0.05 corrected with an FDR. The multivariate ordination technique RDA analyzed loci and environmental predictors simultaneously and determined how loci covary in response to an environmental matrix, detecting strong and weak multilocus associations (Rellstab et al. 2015; Forester et al. 2018). Only common loci detected between GEA methods were considered for further analysis.

### 2.10 Environmental Factors Shaping Genetic Structure

To assess environmental and spatial associations of *F*_*ST*_ outlier loci and the GEA outlier loci datasets, an environmental matrix was built based on ePCA's most significant variables: maximum sea surface temperature (SST_Max), maximum chlorophyll *a* (Chla_Max), and maximum pH (pH_Max). Also, distance‐based Moran's eigenvector maps (*dbMEM*) (Dray et al. 2022) for spatial variables were used, by decomposing Euclidean distance (geographic) using the package ADESPATIAL v.0.0‐8 (Dray et al. 2017). Both loci datasets were evaluated with RDA as implemented in VEGAN v.2.5.6 (Oksanen et al. 2019).

Population minor allele frequency (MAF) of the outlier loci detected by *F*_*ST*_ methods and GEA loci datasets were analyzed, starting with the stepwise ordination method (*ordistep* function) to select correlated variables with statistical significance assessed via *anova.cc* and 999 permutations. Standardized data with the Hellinger method (*decostand* function in the VEGAN package) was used to eliminate broadscale trends. A parsimonious RDA was then performed with the selected variables, and a partial RDA (Borcard, Legendre, and Drapeau 1992) was conducted additionally by conditioning on less significant variables.

### 2.11 Population Assignment and In Silico SNP Power Analysis

Population assignment and in silico SNP power estimations were performed to double cross‐validation was employed to reduce bias in the accuracy of and avoid misleading results (Anderson 2010; Waples 2010). Two datasets were created: one for holding out and one for training, each with an equal number of randomly sampled individuals from previously documented genetic groups (Figure 1). The hold‐out dataset was used to identify the most informative loci, while the training dataset evaluated population assignment accuracy. Each SNP was assessed and ranked using *F*‐statistics in vcftools v.3.0 (Danecek et al. 2011) to optimize genetic differentiation. Seven SNP datasets ranked by FST values (Weir and Cockerham 1984) were generated (50, 100, 200, 300, 500, 800, and 1000 SNPs) and calculated among main groups using the basic.stats function from hierfstat v.0.04–22 (Goudet and Jombart 2015) in R (R Core Team 2014). The performance of each dataset in assigning individuals to populations was evaluated using the R package assignPOP v.1.1.4 (Chen et al. 2018).

Assignment tests for each of the seven datasets were conducted using a MC cross‐validation procedure with the *assign.MC* function from assignPOP. Genotypes from 70% to 80% of individuals from each reporting group were selected as training data, with each combination of training and test datasets iterated 100 times. Predictive models were built using the support vector machine (SVM) classification function, known for its higher assignment accuracies (Chen et al. 2018). To evaluate assignment accuracy, two runs were performed using all available markers: one with all individuals per group and another with only the training dataset. Additional software implementation details are provided by Mejía‐Ruíz et al. (2023).

## 3 Results

### 3.1 SNP Filtering and Datasets

Demultiplexed processing resulted in a dataset of 1,813,283,642 raw reads retained for 303 individual samples, averaging 5.7 million reads each (Tables S1 and S2). Samples with less than one million reads (*n* = 23) and three duplicate individuals (*n* = 3) were discarded, leaving 277 samples for reference genome alignment. A total of 1,277,832,122 primary properly paired alignments were identified using the BWA‐MEM algorithm, and the *ref_map.pl* assembly module in Stacks detected 464,889 SNPs (Tables S3 and S4). The *populations* module was executed for the SNPs filtering process (*r* = 0.8; *min_maf* > 0.03, *write_single_snp*), considering 100% of the populations where a locus must be present to be processed (*p* = 12) and keeping loci with less than 10% of missing data. After the FDR correction, significant linkage disequilibrium detected 470 linked loci. Individuals with > 17% of missing genotypes (*n* = 20) were excluded, which resulted in a global database of 257 individuals and 9117 SNPs (Table S5) contrasting with a previous global database of 175 individuals and 2216 SNPs found by Mejía‐Ruíz et al. (2020) (Table S6).

The *F*_*ST*_ outlier methods Bayescan and PCADAPT detected 132 loci (log PO 0.6) and 76 loci (*q* ≤ 0.05), respectively but only 17 loci were identified by both methods and removed from the global dataset to create the outlier dataset. The HWD test was conducted in the remaining SNPs dataset and no SNPs in disequilibrium across all 12 locations were identified after the sequential Bonferroni correction (*p* ≤ 0.000006). As a result, the neutral dataset consisted of 9100 SNPs and the *F*_*ST*_ outlier dataset of 17 SNPs.

### 3.2 Population Structure

Overall, population structure analyses were consistent among each other for the neutral SNPs datasets. The neutral genetic diversity indexes showed similar values across the species sampling area, with the expected heterozygosity (*He*) ranging from 0.19 to 0.20 (Table 1). The neutral genomic structure identified three clusters: a northern group (DPH, SCnaI, and EN), the Guadalupe Island group, and the coastal BCP locations as a southern group (SJI, FSJ, CI, PE, BT, PuE, BA, and LB). The outliers SNPs dataset showed the same genomic structure.

**TABLE 1:** Genetic diversity parameters for the green abalone ( *Haliotis fulgens* ) along its geographical distribution.

Region	Location	Abrev.	*N*	*Ng*	*He*	*Ho*	*Fis*
Cal	Danna Point Harbor	DPH	39	24	0.19	0.19	0.017
Cal	Santa Catalina Island	SCnaI	27	25	0.19	0.19	0.020
BCP	Ensenada	EN	31	28	0.19	0.19	0.020
BCP	Guadalupe Island	GI	40	29	0.19	0.18	0.052
BCP	San Jeronimo Island	SJI	13	13	0.19	0.18	0.049
BCP	Faro San José	FSJ	12	11	0.19	0.17	0.067
BCP	Cedros Island	CI	25	24	0.20	0.18	0.065
BCP	Punta Eugenia	PE	20	19	0.20	0.19	0.051
BCP	Bahía Tortugas	BT	20	14	0.20	0.19	0.050
BCP	Puerto Escondido	PuE	20	20	0.20	0.19	0.056
BCP	Bahía Asunción	BA	36	32	0.20	0.18	0.061
BCP	La Bocana	LB	20	18	0.20	0.19	0.043
	Total		303	257			

*Note:* Parameters: (*N*) Total samples; (*Ng*) total genotyped samples; (*He*) expected heterozygosity; (*Ho*) observed heterozygosity; (*Fis*) inbreeding coefficient.

Neutral pairwise *FST* analysis first showed a slightly different structure pattern of low values ranging from −0.00013 to 0.0598, grouping undifferentiated values within the California locations (DPH and SCnaI) and the southern group (from CI to LB), but showing significant differences of the BCP northern locations (EN to FSJ) from the rest after sequential Bonferroni correction (*p* ≤ 0.0036; Table S7). The *FST* values of outlier loci were high (range: −0.0169 to 0.4362), highlighting a separation pattern among the northern group (DPH, SCnaI, and EN), Guadalupe Island (GI) and the coastal BCP locations (SJI, FSJ, CI, PE, BT, PuE, BA, and LB) (*p* ≤ 0.0019; Table S8).

Regarding the DAPCs, three clusters were denoted after retaining the first 60 PCs, as suggested by the *xvalDapc* function and the lowest BIC value (Figure S1a,b) for the neutral loci dataset. The first two PCs were retained for the outlier loci dataset, and a single cluster was inferred with BIC (Figure S1c,d). The scatterplot displayed the same three main clusters: (1) the northern group (DPH, SCnaI, and EN), (2) the Guadalupe Island group (GI), and (3) the southern group of coastal BCP locations (SJI, FSJ, CI, PE, BT, PuE, BA, and LB) (Figure 1a,b, respectively).

The Bayesian clustering method of STRUCTURE for the neutral loci dataset indicated *K* = 2 (Figure S2a), and *K* = 3 for the outlier loci dataset (Figure S2b). However, we considered *K* = 3 for the neutral dataset given consistent DAPC previous results. We observed a clear clustering pattern for neutral and outlier datasets, where the northern group (DPH, SCnaI, and EN), the Guadalupe Island group (GI), and the southern group of coastal BCP locations (SJI, FSJ, CI, PE, BT, PuE, BA, and LB) (Figure 1d,e, respectively).

### 3.3 Genetic Connectivity and IBD

Relative directional migration described connectivity patterns consistent with the structuring analyses. In particular, gene flow was observed within the localities of the northern group (DPH, SCnaI, and EN), but not with the rest of the locations of the Baja California Peninsula (BCP), among which a greater gene flow was observed, except for the GI location. However, limited flow was also observed from the northernmost BCP locations (SJI and FSJ), reflecting the slight separation in the DAPC of the neutral dataset (Figure 1c).

The global IBD analysis using 9100 neutral loci and all sampling locations showed genetic and geographic distance are significantly correlated (*r* ^2^ = 0.775, *p* < 0.05) (Figure S3a), as well as when hierarchical IBD (*r* ^2^ = 0.544, *p* < 0.05) after removing the northern group locations (DPH, SCnaI, and EN) (Figure S3b).

### 3.4 Perspective on Genomic–Environment Associations

#### 3.4.1 Seascape Variable Structure and GEA's Marker Detection

**FIGURE 2:**
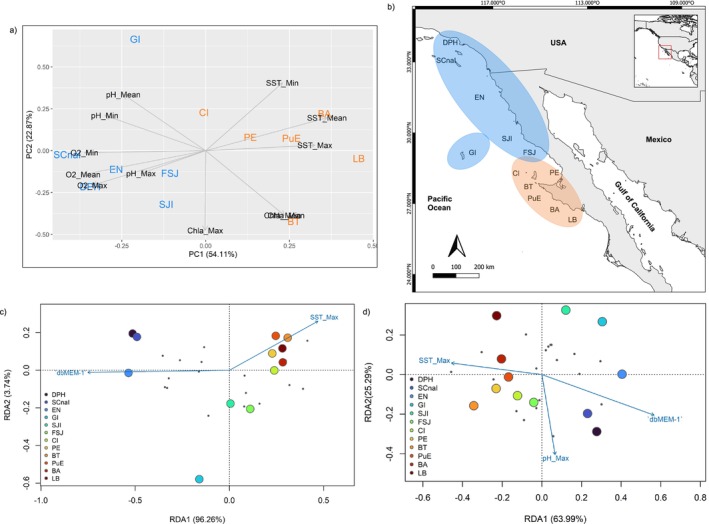
Environmental principal component analysis (ePCA) from sampling locations (a) and its geographic clustering within the area of the California Current System (b). Redundancy analysis (RDA) for (c) 17 *FST* outlier loci; and (d) 19 GEA outlier loci. Arrows are variables significantly related to the population structure: (dbMEM‐1). Circles indicate sampling locations: Dana Point Harbor (DPH), Santa Catalina Island (SCnaI), Ensenada (EN), Guadalupe Island (GI), San Jerónimo Island (SJI), Faro San José (FSJ), Cedros Island (CI), Punta Eugenia (PE), Bahía Tortugas (BT), Puerto Escondido (PuE), Bahía Asunción (BA), and La Bocana (LB).

The ePCA displayed two environmental clusters according to the PC1 axis (Figure 2a): a northern group including DPH, SCnaI, EN, GI, SJI, and FSJ locations, and a southern group of CI, PE, BT, PuE, BA, and LB locations (Figure 2b). Furthermore, the first three PCs retained 86.81% of the cumulative variance (Table S9) for the environmental data from the location polygons.

The most representative environmental variable of the first three PCs was dissolved oxygen (O2_Mean), followed by maximum chlorophyll a (Chla_Max), and maximum pH (pH_Max) (Table S10). However, O2_Mean was highly correlated to every other dissolved oxygen variable and all the sea surface temperature variables (Table S11). Considering this outcome, the O2_Mean variable was substituted by SST_Max for further analyses as SST_Max has a relevant biological relevance and more accurate measure from satellite data.

Regarding the marker detection with genotype–environment association methods, the LFMM and RDA analyses identified 19 outlier loci correlated in common with environmental variables: five loci for SST_Max, and seven for Chla_Max and seven for pH_Max variables, which constituted the GEA outlier loci dataset. Also, from the database of the 19 GEA loci, only three were detected in common with the 17 loci dataset detected by *F*_*ST*_ methods (Table S12).

#### 3.4.2 Environmental Factors Shaping Genetic Structure

The RDA was carried out to evaluate the contribution of environmental variables from ePCA (SST_Max, Chla_Max, and pH_Max) and geographic distance to the two outliers datasets: GEA and *F*_*ST*_ outlier datasets. For the *F*_*ST*_ outlier dataset the *ordistep* function identified only one significant correlation to the maximum sea surface temperature variable (SST_Max; *F* = 3.89, *adjR* ^2^ = 0.20, *p* = 0.0374) and the geographic distance (*dbMEM1*; *F* = 13.904, *adjR* ^2^ = 0.54, *p* = 9.999e−05). Therefore, in the GEA outlier dataset, the *ordistep* function identified significant correlations to the maximum sea surface temperature (SST_Max; *F* = 4.21, *p* = 0.0021), maximum pH (*pH_Max*; *F* = 2.59, *p* = 0.0303; *adjR* ^2^ = 0.27) and dbMEM‐1 (*F* = 2.829, *adjR* ^2^ = 0.14, *p* = 0.0021).

Parsimonious RDAs were constructed for the *F*_*ST*_ outlier dataset using SST_Max as an environment variable and dbMEM‐1 as a spatial variable, and for the GEA outlier dataset SST_Max, pH_Max, and dbMEM‐1. The parsimonious RDA for *F*_*ST*_ outlier loci dataset detected a significant correlation with predictor variables (*F* = 10.727, *adjR* ^2^ = 0.64, *p* = 0.001), where the first RDA axis explained 96.26% and the second axis 3.74% (*adjR* ^2^ = 0.6387) of the model variance (Figure 2C). Similarly, the parsimonious RDA for GEA outlier loci dataset found a significant correlation with predictor variables (*F* = 3.736, *adjR* ^2^ = 0.43, *p* = 0.001), where the first RDA axis explained 63.99% and the second RDA axis 25.29 of the model variance (Figure 2d). However, partial RDA for the *FST* outlier loci only found a significant correlation for the spatial variable of geographic distance (dbMEM‐1) when controlling for the environmental variable of maximum sea surface temperature (SST_Max) (*F* = 9.4692, *adjR* ^2^ = 0.3923, *p* = 0.0009) (Figure S4). Partial RDA for the GEA outlier loci significant correlation was found for the environmental variables SST_Max and pH_Max (*F* = 2.964, *adjR* ^2^ = 0.2418, *p* = 0.0009) when controlling for the spatial variable of geographic distance (dbMEM‐1) (Figure S5). Hence, results of IBD and outlier loci datasets indicate that geographic distance plays a major role in shaping genomic diversity and evidence of environmental effect remains limited with the current dataset.

### 3.5 Population Assignment and In Silico SNP Power Analysis

The global data set included a total of 9117 loci and the three groups: (1) North (DPH, SCnaI, and EN), (2) Guadalupe Island (GI), and (3) South (SJI, FSJ, CI, PE, BT, PuE, BA, and LB).

**FIGURE 3:**
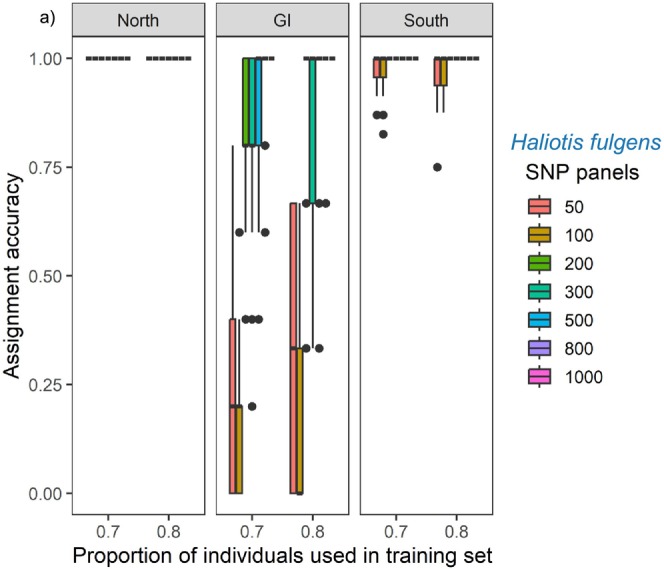
Assignment accuracies estimated via Monte Carlo cross‐validation and SVM methods, with two levels of training individuals (0.7 and 0.8) from the training dataset (*n* = 52), and eight panels of SNPs (50–1000 most differentiated SNPs). Box plot details: The line within the box is the median; the top and bottom edges of the box are 25 and 75th percentiles; the ends of whiskers are the minimum and maximum of non‐outliers, and outliers are shown as black dots.

After MC cross‐validation and the SVM method, overall assignment accuracies exhibited high discriminatory power (> 75%) mainly for every SNP panel of the North and South groups. Individuals from the North group (DPH, SCnaI, and EN) had the highest assignment rates for every SNP panel. Individuals from the South group (SJI, FSJ, CI, PE, BT, PuE, BA, and LB) ranged from 90% to 100% for the 50 and 100 SNP panels but showed a 100% rate for the rest of the SNP panels. In contrast, individuals from GI showed the lowest assignment accuracies for the 50 and 100 SNP panels ranging from 0% to 70%, followed by the 200, 300, and 500 SNP panels ranging from 70% to 100%, nonetheless, the 800 and 1000 SNP panels exhibited a 100% assignment accuracy rate (Figure 3; Tables S12 and S13).

## 4 Discussion

This study provides information on the green abalone genetic structure and connectivity at an international scale to understand the population dynamics and inform conservation strategies between the United States and Mexico (CDFGM, 2005; DOF 2023). Evidence on neutral genomic structure and genetic connectivity reveals a dominant role over the potential local adaptations in green abalone, suggesting that geographic distance and life history may influence patterns of genetic structuring. For green abalone, there is insufficient evidence of genome–environment associations for adaptive structure responding to environmental pressure, which is indistinguishable from the neutral structure, in contrast with local adaptations described for pink abalonein the same geographic distribution, rejecting the initial hypothesis of a marked influence of natural selection based on Marine Ecoregions (Mares‐Mayagoitia et al. 2024). Therefore, the current study focuses mainly on discussing the results obtained from the neutral genomic structure of green abalone.

### 4.1 Population Genomic Structure and Potential Adaptive Groups

The analysis of neutral genomic structure revealed three distinct populations: the North group covering California, USA, to Ensenada, Baja California (DPH, SCnaI, and EN), the Guadalupe Island (GI) group, and the South group, including the coastal locations of the BCP (SJI, FSJ, CI, PE, BT, PuE, BA, and LB).

The North group delimitates geographically from California to Ensenada, Baja California, representing a relevant finding, as it clearly shows a structure and genetic connectivity between locations in the United States and Mexico not previously observed for this species, nor the pink abalone with whom it shares distribution range (Díaz‐Viloria et al. 2009; Munguía‐Vega et al. 2015; Mares‐Mayagoitia et al. 2024). The isolated population of Guadalupe Island has been highlighted in previous studies of this species using SNPs (Mejía‐Ruíz et al. 2020) or microsatellites (Gutiérrez‐González et al. 2007). The major contrast between the neutral structure identified by Mejía‐Ruíz et al. (2020) is the subtle clustering of the SJI and FSJ locations classified as part of their northern group, while in this study, such locations are grouped alongside other southern peninsula locations. However, an apparent partitioning of SJI and FSJ locations as another cluster was plausibly observed in the DAPC, raising the question of whether the clustering pattern could be clarified with increased sampling effort in the area between Ensenada, Baja California, and the SJI location. This prompts consideration for future research to explore additional sampling in the area.

The neutral structure and the relative migration rate observed in green abalone appear to be attributed to specific traits of its life cycle (Vélez‐Arellano et al. 2020) and potential larval dispersal characteristics previously estimated in the region for abalone and other marine species (Arafeh‐Dalmau et al. 2023). This pattern mirrors findings across various regions globally, which have identified neutral genetic structure and pronounced connectivity (Dalongville et al. 2018; Muir et al. 2020; Macleod et al. 2024), predominantly shaped by factors such as the impact of ocean currents on larval dispersal (Coscia et al. 2020) and the duration of the larval phase (Dibattista et al. 2017).

Moreover, groups with local adaptation displayed the same pattern as the neutral structure, differing from the results described for pink abalone (Mares‐Mayagoitia et al. 2024). Similarities between neutral and adaptive structure patterns have been recorded in previous studies, such as the case of the giant black tiger shrimp ( *Penaeus monodon* ) throughout the Indo‐Pacific Region (Vu et al. 2021), and the blenny ( *Eleginops maclovinus* ) from north Patagonia (Canales‐Aguirre et al. 2022).

However, no significant genotype–environment association was found in the green abalone, suggesting that this structure may be primarily influenced by geographic distance and, possibly, by marine current patterns. Another potential explanation for the lack of genotype–environment association might be related to the reduced genomic coverage of this study, since, fragment selection in library preparation was tight (± 50 pb). There is evidence that a sister species, black abalone ( *Haliotis cracherodii* ), presents a large extent of spatial and genome‐wide homogeneity and a single linkage group that presents an inversion where it might natural selection acts (Wooldridge et al. 2024). Whether green abalone also presents a limited number of linkage groups under selection, it is necessary to develop a genome‐wide study to acquire an in‐depth insight into the genetic diversity of green abalone.

### 4.2 Genetic Connectivity Overlays the Signal of Local Adaptations

The main motivation of the current research was to investigate a hypothesis based on the findings by Stanley et al. (2018), which exhibited that an environmental gradient, specifically sea surface temperature, is correlated to potential adaptative groups among different species with distinct life cycles in the Atlantic coastal borderline between the United States and Canada.

To address this hypothesis, the green abalone was an interesting model species given the shared traits with the pink abalone, such as spatial distribution, and inhabits the CCS. In this area, Spalding et al. (2007) described two Marine Ecoregions with environmental characteristics delimited to the north (Southern California Bight) and south (Magdalena Transition) of Punta Eugenia on the BCP, highlighting an environmental gradient denoting cold and warm waters, respectively. However, we did not uncover the expected pattern proposed by Stanley et al. (2018), and the disparity in responses of green and pink abalone related to the environmental gradient highlights the complex interplay between environmental factors and species‐specific characteristics in shaping genetic connectivity and adaptive grouping.

The IBD and RDA analyses revealed that geographic distance plays a pivotal role in shaping neutral and adaptive genetic structures respectively, in green abalone, as opposed to the patterns observed in pink abalone. Detecting geographic distance as a significant variable underscores the importance of considering spatial dynamics in understanding the genetic structure of marine species alongside differences in species‐specific life cycles.

The genetic structure can vary significantly even between species within the same family, and differences in reproductive strategies and life cycles can contribute to these variations (van der Ven et al. 2021; Buitrago‐López et al. 2023). Green abalone occurs from intertidal to ~24 m depth and pink abalone from 7 to 44 m water depth (Sierra‐Rodríguez et al. 2006). According to Vélez‐Arellano et al. (2020), green and pink abalone are broadcast spawners with different reproductive seasons; green abalone spawning has been reported in autumn–winter and winter–spring, while pink abalone can reproduce throughout the year, given the presence of favorable environmental conditions. These differences in reproductive modes can influence patterns of genetic differentiation in terms of gene flow as green abalone might show highly localized recruitment due to a shallower bathymetric distribution resulting in restricted connectivity, compared to deeper spawning of pink abalone and larval dispersal influenced by stronger marine currents.

The influence of different life histories on genetic and genomic structure is illustrated by other invertebrate groups, such as corals (Harrison and Wallace 1990). For instance, broadcast spawning corals *Acropora digitifera* and *Pocillopora verrucosa* formed a single panmictic population characterized by extensive gene flow, while brooding corals *Isopora brueggemanni* and *Stylophora pistillata* displayed high levels of genetic structure (Thomas et al. 2020; Buitrago‐López et al. 2023).

### 4.3 Research Scope

The green abalone reference genome allowed us to increase the number of SNPs from 2216 obtained by the *denovo* genotyping method (Mejía‐Ruíz et al. 2020), to 9117, representing 99.63% of successfully mapped alignments, and accentuating the difference from that obtained by a non‐model species phylogenetically close at genus level such as the pink abalone, which showed 97.18% of successfully mapped alignments (Mares‐Mayagoitia et al. 2024). In addition, the strict parameters used during the loci filtering process (−*r*: 80% of individuals; −*p*: 100% of sampled locations), favored the retention of loci with values below 10% of missing data per individual and less than 17% per loci, enhancing the statistical power of our SNPs and reducing the potential batch effect between California and Mexico genomic libraries (Rochette and Catchen 2017).

The main result of this study points to a significant influence of geographic distance on genetic connectivity, overshadowing the role of natural selection. The influence of ocean currents on larval dispersal and their effect on gene flow remains unknown. This is a significant limitation for integrating genetic data with ocean circulation modeling. Larval dispersal simulations would allow for unraveling the intricate dynamics of genetic connectivity among green abalone populations, ultimately contributing to more effective marine conservation and management strategies (Jahnke and Jonsson 2022). By accurately simulating ocean currents and larval dispersal patterns, researchers gain insights into how various factors such as geography, bathymetry, and hydrodynamics influence the movement of larvae and subsequent gene flow (Treml et al. 2015). For instance, Galaska et al. (2021) employed a combination of seascape genomic analyses with a 4248 neutral SNP dataset, precise ocean circulation modeling, and larval dispersal simulations to measure population structure and connectivity within the octocoral *Paramuricea biscaya*, identifying that genetic structure is influenced by depth and that larval dispersal between genetic interconnected populations is uneven owing to prevailing ocean circulation patterns. Considering all this, we are only one step away from more precisely understanding the interaction between larval dispersal and genetic connectivity of green abalone.

### 4.4 Future Directions in Conservation and Management Strategy

In the central region of the Baja California Peninsula there are success stories for the conservation and management of marine resources, mainly focused on the establishment of marine reserves by cooperative societies formed by local fishermen in which a positive trend has been observed in the recovery of species in and around protected areas (Micheli et al. 2012; Smith et al. 2022). Although these measures might be in the early stages and at a local scale, they are of utmost importance because they reflect the interest of local fishers in the conservation and proper management of their resources.

Thus, the results of population assignment analysis in this study represent the first step in developing new strategies that contribute to the conservation and management of fishery resources. The set of SNP markers that allows a correct assignment close to 100% for the three identified green abalone populations represents 8.77% of a total of 9117 SNPs, so developing a panel of markers for traceability would be the steps to follow to help ensure the sustainability and legality of seafood supply chains, ultimately benefiting both fishers and consumers (Aranceta‐Garza, Pérez‐Enríquez, and Cruz 2011). In addition, it would also favor translocation efforts through genetic monitoring as has been previously attempted in Mexico (Searcy‐Bernal et al. 2013) and the United States (Taniguchi et al. 2013), strengthening binational relationships on conservation and management issues.

Furthermore, it is necessary to consider and propose an interdisciplinary collaborative strategy, as in the case evaluated by Moreno et al. (2017) in the Mexican Caribbean, where an initiative called “Kanan Kay Alliance” emerged and built a voluntary multi‐stakeholder collaborative network of more than 40 organizations, including fishing cooperatives, government, regional NGO's, researchers and philanthropic foundations. Therefore, transferring and translating such experience to this region with a binational perspective would be a new challenge.

## 5 Conclusions

This study reveals that neutral genetic structure and geographic distance play pivotal roles in shaping population dynamics, masking the effect of local adaptations and the need for more in‐depth studies to identify genotype–environment associations to promote comprehensive conservation strategies spanning Mexico and the United States borders. However, a genome‐wide study is necessary to address the potential limitations of the present study and clarify the genome‐wide effects of natural selection.

Despite its similar distribution with the pink abalone, the genetic structure of green abalone shows distinct patterns, particularly in the delineation of populations between the United States and Mexico. Our findings emphasize the need to study the larval dispersal characteristics and life history differences in genetic structure, given that population management strategies should account for neutral and adaptive genetic variation to conserve green abalone populations effectively.

This study points to the need for international collaboration to develop effective conservation and management strategies for green abalone populations. Drawing from successful initiatives in other regions, such as the establishment of marine reserves by local fishing cooperatives, future efforts should prioritize the development of SNP markers for traceability and genetic monitoring to support translocation efforts and ensure the sustainability of seafood supply chains.

## Author Contributions

**Jorge Alberto Mares‐Mayagoitia:** conceptualization (equal), data curation (equal), formal analysis (equal), investigation (equal), methodology (equal), writing – original draft (lead), writing – review and editing (lead). **Paulina Mejía‐Ruíz:** conceptualization (equal), data curation (equal), formal analysis (equal), investigation (equal), methodology (equal), writing – original draft (equal), writing – review and editing (equal). **Fabiola Lafarga‐De la Cruz:** conceptualization (supporting), data curation (supporting), formal analysis (supporting), investigation (supporting), writing – original draft (supporting), writing – review and editing (supporting). **Fiorenza Micheli:** writing – review and editing (supporting). **Pedro Cruz‐Hernández:** conceptualization (supporting), data curation (supporting), investigation (supporting), writing – review and editing (supporting). **Juan A. De‐Anda‐Montañez:** conceptualization (supporting), formal analysis (supporting), funding acquisition (supporting), methodology (supporting), project administration (supporting), writing – review and editing (supporting). **John Hyde:** data curation (supporting), funding acquisition (supporting), investigation (supporting), resources (supporting), writing – review and editing (supporting). **Norma Y. Hernández‐Saavedra:** conceptualization (supporting), funding acquisition (supporting), investigation (supporting), project administration (supporting), resources (supporting), writing – review and editing (supporting). **Vladimir S. De Jesús‐Bonilla:** conceptualization (supporting), data curation (supporting), formal analysis (supporting), investigation (supporting), methodology (supporting), writing – review and editing (supporting). **Carmen E. Vargas‐Peralta:** data curation (supporting), formal analysis (supporting), investigation (supporting), methodology (supporting), project administration (supporting), resources (supporting), writing – review and editing (supporting). **Ana L. Flores‐Morales:** data curation (supporting), formal analysis (supporting), methodology (supporting), writing – review and editing (supporting). **Alejandro F. Pares‐Sierra:** data curation (supporting), formal analysis (supporting), methodology (supporting), writing – review and editing (supporting). **Fausto Valenzuela‐Quiñonez:** conceptualization (lead), data curation (equal), formal analysis (equal), funding acquisition (lead), investigation (lead), methodology (equal), project administration (lead), resources (lead), supervision (lead), writing – original draft (equal), writing – review and editing (equal).

## Conflicts of Interest

The authors declare no conflicts of interest.

## Supporting information


Appendix S1.

## Data Availability

Raw sequence reads are available in NCBI's Sequence Read Archive (SRA) under accession number PRJNA1185316 (SRR31321040‐SRR31321292). Scripts and databases will be available in DRIAD: https://doi.org/10.5061/dryad.m37pvmdcm.
