# Supplementary material for: A Seascape Genomics Perspective on Restrictive Genetic Connectivity Overcoming Signals of Local Adaptations in the Green Abalone (Haliotis fulgens) of the California Current System
Source: Ecol Evol. 2025 Feb 4;15(2):e70913. doi: 10.1002/ece3.70913 (PMC11794835; doi:10.1002/ece3.70913)
Supplement: Supplementary file 1 — Appendix S1. [file ECE3-15-e70913-s001.docx]

# A seascape genomics perspective on restrictive genetic connectivity overcoming signals of local adaptations in the green abalone (*Haliotis fulgens*) of the California Current System.

**SUPPLEMENTARY FIGURES**


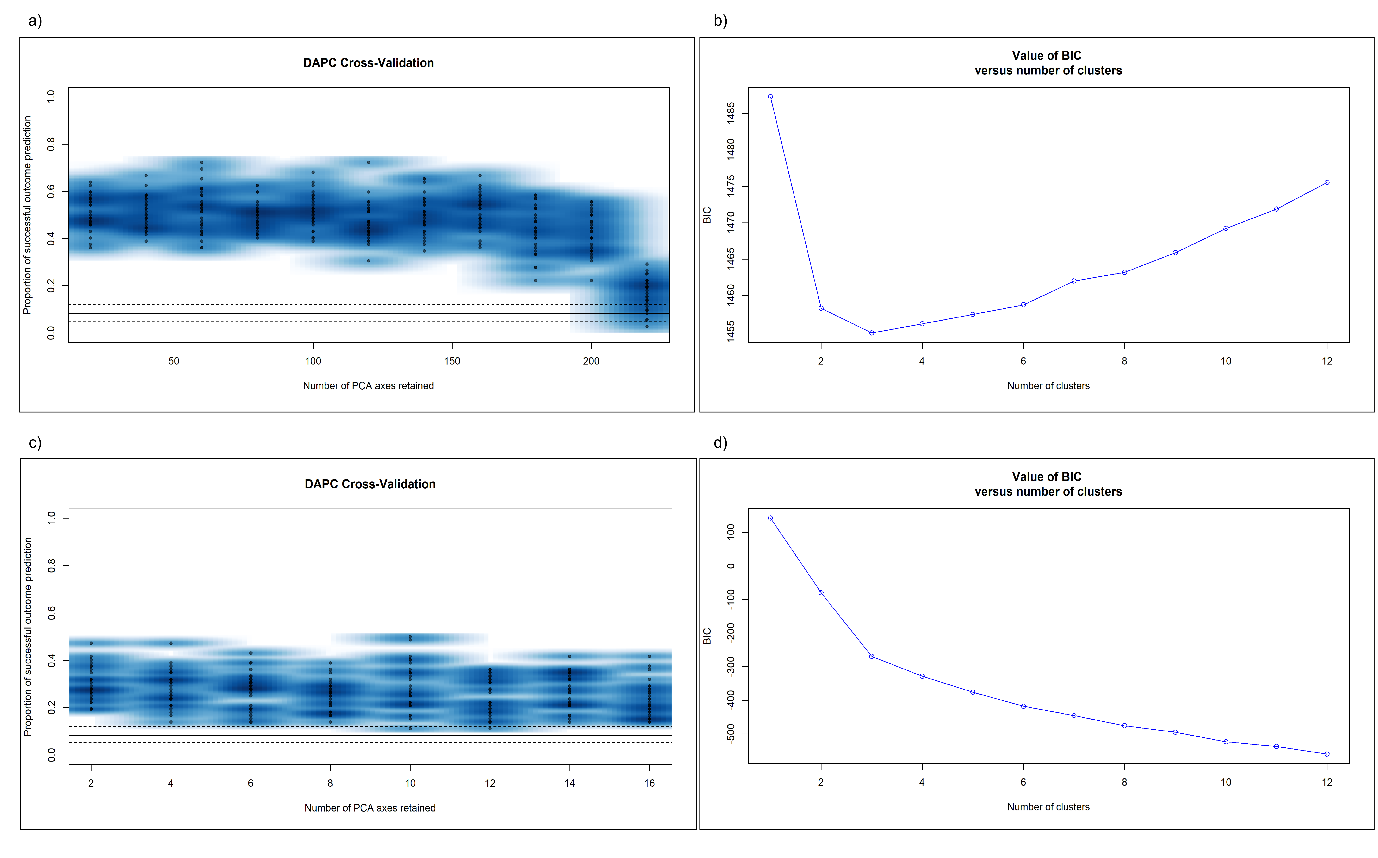


**Supplementary Figure S1.** DAPC Cross validation and BIC tests for 9,100 neutral loci (**a** and **b**), and 17 outliers’ dataset (**c** and **d**).


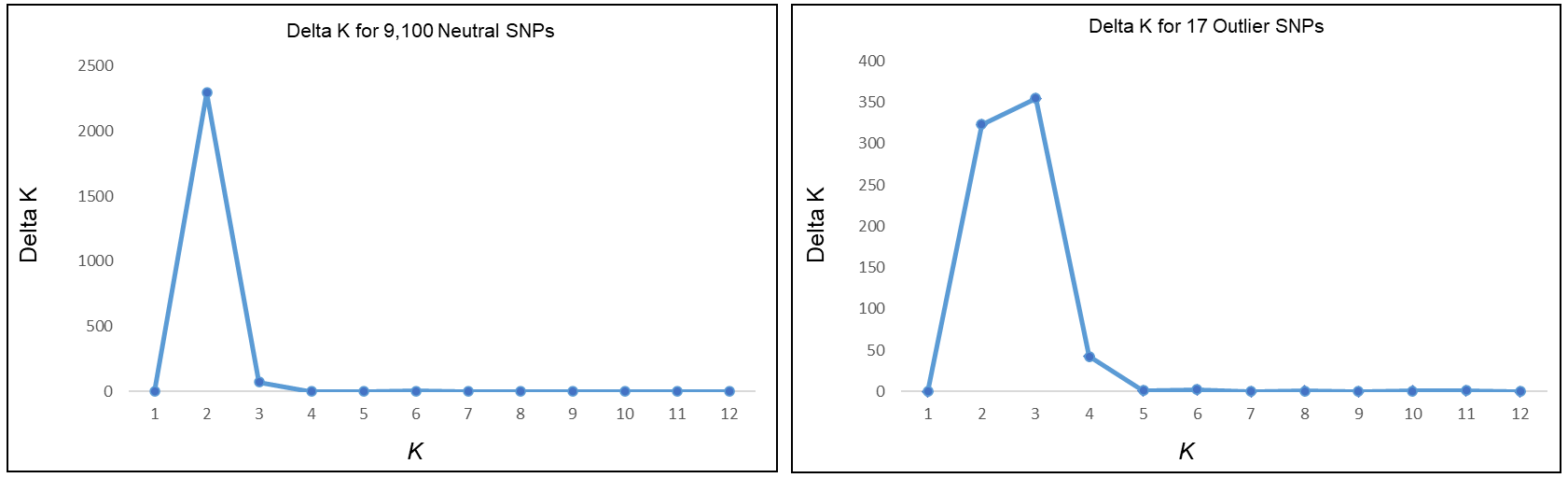


**Supplementary Figure S2.** Evanno delta *K* test for the **(a)** 9,100 neutral SNPs and **(b)** 17 outliers’ datasets from STRUCTURE.

**
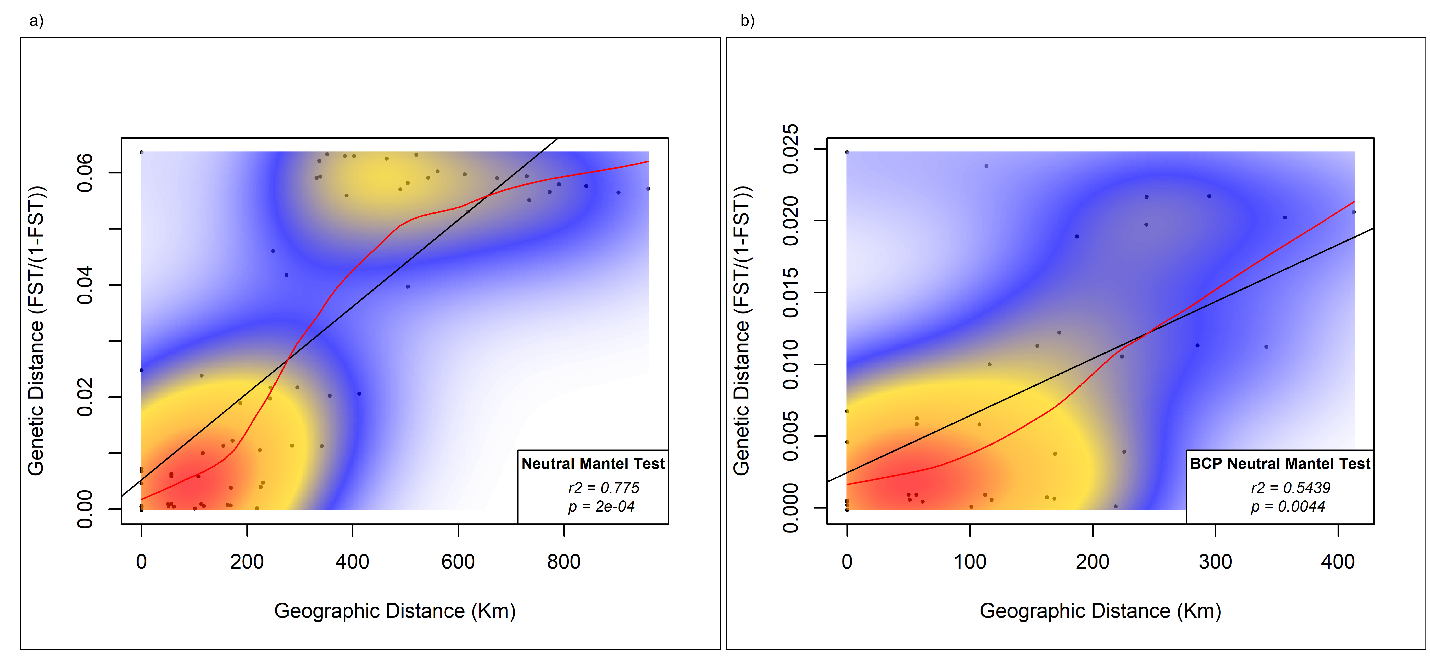
**

**Supplementary Figure S3.** Isolation by Distance (IBD) analysis for the green abalone (*Haliotis fulgens*) contrasting 9,100 neutral SNPs for the total sampled locations **(a)** and hierarchical IBD with BCP locations (**b**).

**
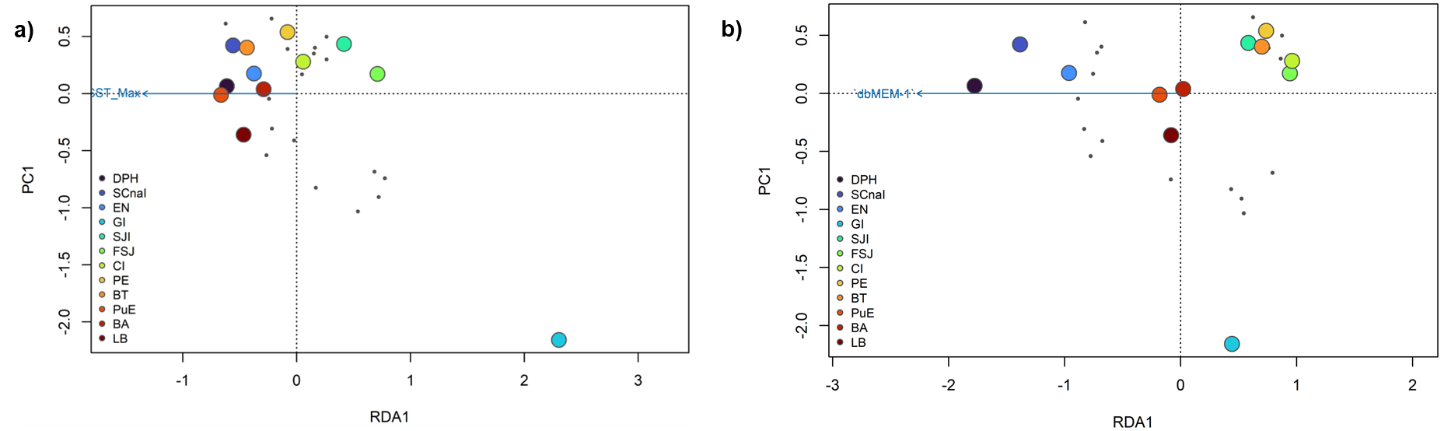
**

**Supplementary Figure S4.** Partial RDA of the 17 outlier by *F_ST_* loci dataset when controlling for the spatial variable “dbMEM-1” (**a**; *p* = 0.5085, *F* = 0.934, *adjR^2^* = -0.003), and controlling for “SST_Max” (**b**; *p* = 0.0009, *F* = 9.469, *adjR^2^* = 0.39). Arrows are variables significantly related to the population structure (**a**). Circles indicate sampling locations: Dana Point Harbor (DPH), Santa Catalina Island (SCnaI), Ensenada (EN), Guadalupe Island (GI), San Jerónimo Island (SJI), Faro San José (FSJ), Cedros Island (CI), Punta Eugenia (PE), Bahía Tortugas (BT), Puerto Escondido (PuE), Bahía Asunción (BA) and La Bocana (LB).

**
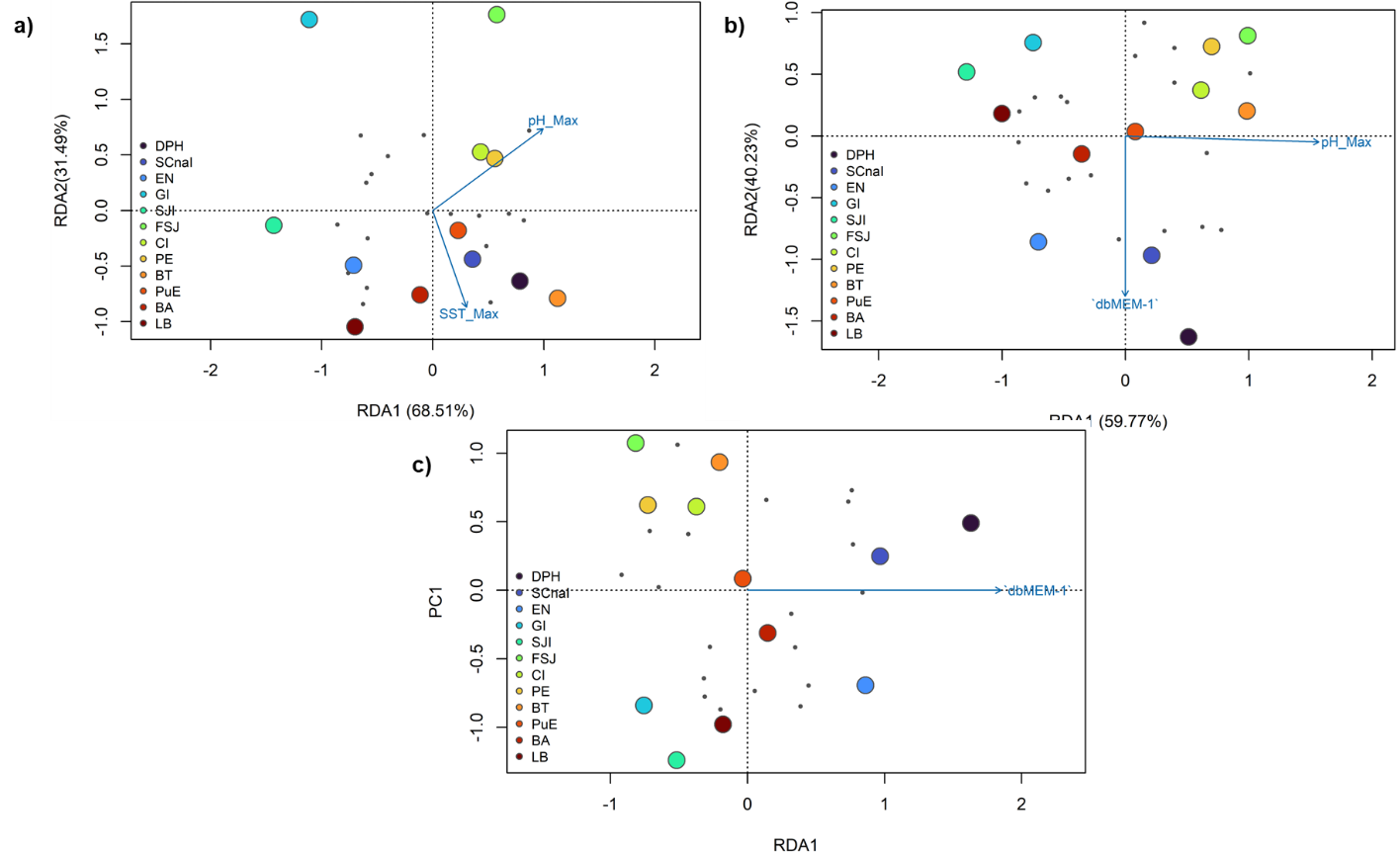
**

**Supplementary Figure S5.** Partial RDA of the 19 GEA loci dataset when controlling for the spatial variable “dbMEM-1” (**a**; *p* = 0.0009, *F* = 2.964, *adjR^2^* = 0.24); controlling for “SST_Max” (**b**; *p* = 0.0009, *F* = 3.258, *adjR^2^* = 0.27), and controlling for “SST_Max” and “pH_Max” (**c**; *p* = 0.0609, *F* = 1.983, *adjR^2^* = 0.08). Arrows are variables significantly related to the population structure (**a**). Circles indicate sampling locations: Dana Point Harbor (DPH), Santa Catalina Island (SCnaI), Ensenada (EN), Guadalupe Island (GI), San Jerónimo Island (SJI), Faro San José (FSJ), Cedros Island (CI), Punta Eugenia (PE), Bahía Tortugas (BT), Puerto Escondido (PuE), Bahía Asunción (BA) and La Bocana (LB).

**SUPPLEMENTARY TABLES**

**Supplementary Table S1.** Demultiplexing summary statistics.

| **Demultiplex** | **Total** |
| --- | --- |
| Total Sequences | 2,108,074,272 |
| Barcode Not Found | 18,888,048 |
| Low Quality | 207,868,637 |
| RAD Cutside Not Found | 68,031,889 |
| Retained reads | **1,813,283,642** |

**Supplementary Table S2.** Every individual data from raw sequence to properly paired alignment.

| **Hf Ind ID** | **Indx_sec** | **Barcode** | **Location** | **Raw-Seq** | **Retained** | **Alignments** |
| --- | --- | --- | --- | --- | --- | --- |
| AZCBP_1 | ATCACG | CTGTC | DPH | 3,549,654 | 2,963,016 | 1,997,123 |
| AZCBP_2 | ATCACG | CTTGG | DPH | 2,773,844 | 2,324,302 | 1,569,896 |
| AZCBP_5 | ATCACG | GAGTC | DPH | 3,424,512 | 2,913,928 | 2,013,191 |
| AZCBP_9 | ATCACG | GGATA | DPH | 4,079,234 | 3,440,777 | 2,368,407 |
| AZCBP12 | ATCACG | GGCTC | DPH | 2,832,644 | 2,404,410 | 1,658,067 |
| AZCBP17 | TGACCA | TAGTA | DPH | 9,680,696 | 7,526,225 | 4,609,868 |
| AZCBP18 | CTTGTA | GCATG | DPH | 10,669,780 | 8,599,355 | 5,730,597 |
| AZCBP19 | CTTGTA | AACCA | DPH | 7,367,198 | 5,920,648 | 3,801,375 |
| AZCBP23 | CTTGTA | TCGAT | DPH | 4,560,164 | 3,722,703 | 2,440,570 |
| AZCBP24 | CTTGTA | TGCAT | DPH | 6,680,474 | 5,375,775 | 3,480,574 |
| AZCDP_2 | ATCACG | ATGAG | DPH | 4,957,834 | 4,171,283 | 2,809,448 |
| AZCDP_3 | ATCACG | ATTAC | DPH | 7,101,396 | 6,005,165 | 4,181,934 |
| AZCDP_4 | ATCACG | CATAT | DPH | 5,357,690 | 4,546,505 | 3,164,760 |
| AZCDP_6 | ATCACG | CGAAT | DPH | 4,264,044 | 3,563,475 | 2,404,803 |
| AZCDP_7 | ATCACG | CGGCT | DPH | 4,255,272 | 3,566,763 | 2,452,498 |
| AZCDP_8 | ATCACG | CGGTA | DPH | 5,448,040 | 4,537,424 | 3,090,729 |
| AZCDP_9 | ATCACG | CGTAC | DPH | 4,561,498 | 3,827,811 | 2,628,393 |
| AZCDP11 | ATCACG | CTGAT | DPH | 3,943,068 | 3,356,874 | 2,350,646 |
| AZCDP18 | ACTTGA | AATTA | DPH | 7,250,224 | 5,561,534 | 3,406,097 |
| AZCDP19 | CAGATC | ATGAG | DPH | 8,756,524 | 6,798,305 | 4,193,607 |
| AZCDP21 | CAGATC | CATAT | DPH | 2,732,620 | 2,068,880 | 1,230,285 |
| AZCDP22 | CAGATC | CGGTA | DPH | 8,495,178 | 6,153,481 | 3,440,336 |
| AZCDP23 | CAGATC | CGTAC | DPH | 6,843,214 | 5,194,153 | 3,107,848 |
| AZCDP24 | CAGATC | CTGAT | DPH | 9,438,944 | 7,374,622 | 4,624,345 |
| AZCWA_1 | TTAGGC | CGATC | SCnaI | 3,450,494 | 2,613,599 | 1,626,397 |
| AZCWA_2 | TTAGGC | TCGAT | SCnaI | 12,083,778 | 9,572,893 | 6,135,090 |
| AZCWA_3 | TTAGGC | TGCAT | SCnaI | 6,636,588 | 5,223,117 | 3,291,021 |
| AZCWA_5 | TTAGGC | GGTTG | SCnaI | 8,416,822 | 6,687,509 | 4,209,462 |
| AZCWA_7 | TTAGGC | AAGGA | SCnaI | 7,634,992 | 5,946,274 | 3,753,840 |
| AZCWA_8 | CGATGT | GAGAT | SCnaI | 5,614,684 | 4,595,309 | 3,096,043 |
| AZCWA_9 | CGATGT | GAGTC | SCnaI | 7,113,894 | 5,916,744 | 4,058,035 |
| AZCWA10 | CGATGT | GCCGT | SCnaI | 7,520,360 | 6,225,829 | 4,228,993 |
| AZCWA11 | CGATGT | GCTGA | SCnaI | 11,292,914 | 9,359,881 | 6,448,915 |
| AZCWA12 | CGATGT | GGATA | SCnaI | 4,644,170 | 3,848,165 | 2,603,785 |
| AZCWA13 | CGATGT | GGCCA | SCnaI | 10,708,714 | 8,908,549 | 6,117,960 |
| AZCWA14 | CGATGT | GGCTC | SCnaI | 8,471,686 | 7,037,878 | 4,821,851 |
| AZCWA15 | CGATGT | GTAGT | SCnaI | 4,342,944 | 3,608,124 | 2,405,926 |
| AZCWA16 | CTTGTA | CTGCG | SCnaI | 9,848,766 | 7,393,456 | 4,345,162 |
| AZCWA19 | TAGCTT | CTGTC | SCnaI | 6,025,260 | 4,484,113 | 2,600,574 |
| AZCWA20 | TAGCTT | CTTGG | SCnaI | 11,962,948 | 9,154,118 | 5,529,402 |
| AZCTB_1 | CGATGT | CTTGG | SCnaI | 3,362,914 | 2,843,023 | 1,983,477 |
| AZCTB_2 | CGATGT | GACAC | SCnaI | 3,265,292 | 2,768,384 | 1,929,177 |
| AZCTG_1 | TGACCA | TCCGG | SCnaI | 10,692,618 | 5,827,240 | 2,976,776 |
| AZCTG_3 | TGACCA | TGGAA | SCnaI | 4,416,766 | 3,549,128 | 2,334,062 |
| AZCTG_4 | TGACCA | TTACC | SCnaI | 4,374,580 | 3,575,728 | 2,367,698 |
| AZCTG_5 | TTAGGC | GCATG | SCnaI | 3,486,914 | 2,846,360 | 1,916,487 |
| AZCTG_6 | TTAGGC | AACCA | SCnaI | 2,902,602 | 2,371,085 | 1,625,169 |
| AZCTG_7 | CGATGT | CTGCG | SCnaI | 3,475,412 | 2,940,745 | 2,058,417 |
| AZCTG_8 | CGATGT | CTGTC | SCnaI | 3,154,000 | 2,678,631 | 1,864,902 |
| AZEN_3 | TGACCA | GCTGA | EN | 5,680,970 | 4,280,792 | 2,591,705 |
| AZEN_4 | TGACCA | GGATA | EN | 6,168,994 | 4,625,935 | 2,801,678 |
| AZEN_5 | TGACCA | GGCCA | EN | 9,442,136 | 7,076,365 | 4,169,913 |
| AZEN_6 | TGACCA | GGCTC | EN | 11,755,526 | 8,816,676 | 5,215,313 |
| AZEN_7 | TGACCA | GTAGT | EN | 9,583,236 | 7,247,182 | 4,392,801 |
| AZEN_8 | TGACCA | GTCCG | EN | 12,643,304 | 9,580,861 | 5,744,431 |
| AZEN_9 | TGACCA | GTCGA | EN | 11,758,866 | 8,889,541 | 5,336,710 |
| AZEN10 | CGATGT | AATTA | EN | 5,273,800 | 4,371,278 | 3,010,647 |
| AZEN11 | CGATGT | ACGGT | EN | 5,265,272 | 4,366,319 | 2,987,922 |
| AZEN12 | CGATGT | ACTGG | EN | 6,518,354 | 5,419,354 | 3,733,103 |
| AZEN13 | CGATGT | ACTTC | EN | 8,633,470 | 7,185,714 | 4,945,909 |
| AZEN14 | CGATGT | ATACG | EN | 9,738,162 | 8,116,852 | 5,615,701 |
| AZEN15 | CGATGT | ATGAG | EN | 20,941,864 | 17,596,809 | 12,143,104 |
| AZEN16 | CGATGT | ATTAC | EN | 6,929,234 | 5,750,979 | 3,949,619 |
| AZEN17 | CGATGT | CATAT | EN | 5,621,480 | 4,650,492 | 3,154,187 |
| AZEN18 | CGATGT | CGAAT | EN | 6,549,420 | 5,396,130 | 3,668,456 |
| AZEN19 | CGATGT | CGGCT | EN | 5,393,784 | 4,401,503 | 2,988,582 |
| AZEN20 | CGATGT | CGGTA | EN | 6,013,492 | 4,864,600 | 3,251,151 |
| AZEN21 | CGATGT | CGTAC | EN | 8,392,838 | 6,922,047 | 4,731,273 |
| AZEN23 | CGATGT | CTGAT | EN | 17,944,616 | 14,979,319 | 10,371,325 |
| AZEN24 | ATCACG | TGGAA | EN | 8,782,070 | 7,315,001 | 4,971,511 |
| AZEN25 | ATCACG | TTACC | EN | 6,955,498 | 5,822,939 | 3,959,006 |
| AZEN26 | TGACCA | CGGCT | EN | 16,831,828 | 12,632,535 | 7,531,413 |
| AZEN27 | TGACCA | CGGTA | EN | 13,263,914 | 9,774,736 | 5,566,318 |
| AZEN29 | TGACCA | CGTCG | EN | 11,426,344 | 8,679,169 | 5,188,927 |
| AZEN30 | TGACCA | CTGAT | EN | 5,008,654 | 3,732,874 | 2,122,772 |
| AZEN31 | TGACCA | CTGCG | EN | 7,579,222 | 5,669,248 | 3,338,820 |
| AZEN32 | TGACCA | CTGTC | EN | 9,553,432 | 7,221,635 | 4,337,370 |
| AZIG_4 | TGACCA | ACTGG | GI | 3,722,350 | 2,803,194 | 1,660,771 |
| AZIG11 | CGATGT | GCATG | GI | 2,994,754 | 2,424,188 | 1,558,347 |
| AZIG12 | CGATGT | AACCA | GI | 3,672,408 | 2,975,809 | 1,974,777 |
| AZIG14 | CGATGT | TCGAT | GI | 3,646,838 | 2,975,487 | 1,970,495 |
| AZIG15 | CGATGT | TGCAT | GI | 2,362,056 | 1,934,533 | 1,288,004 |
| AZIG16 | CGATGT | CAACC | GI | 1,826,790 | 1,484,097 | 969,802 |
| AZIG17 | CGATGT | GGTTG | GI | 2,334,518 | 1,930,990 | 1,282,429 |
| AZIG19 | CGATGT | AGCTA | GI | 3,752,578 | 3,090,614 | 2,078,239 |
| AZIG20 | CGATGT | ACACA | GI | 4,240,052 | 3,509,079 | 2,373,811 |
| HF276 | ACAGTG | TCACG | GI | 9,862,170 | 8,934,600 | 6,658,292 |
| HF277 | ACAGTG | TCAGT | GI | 14,929,072 | 13,640,595 | 10,009,182 |
| HF278 | ACAGTG | TCCGG | GI | 16,258,246 | 12,565,471 | 7,498,846 |
| HF279 | ACAGTG | TCTGC | GI | 16,446,274 | 14,962,269 | 11,012,811 |
| HF282 | ACAGTG | TGGAA | GI | 7,608,342 | 6,784,869 | 4,883,308 |
| HF283 | ACAGTG | TTACC | GI | 10,223,944 | 9,265,278 | 6,846,530 |
| HF284 | GCCAAT | GCATG | GI | 13,688,334 | 12,541,008 | 9,311,368 |
| HF285 | GCCAAT | AACCA | GI | 12,561,818 | 11,187,047 | 8,055,298 |
| HF286 | GCCAAT | CGATC | GI | 10,964,702 | 10,094,553 | 7,419,377 |
| HF287 | GCCAAT | TCGAT | GI | 13,536,554 | 12,539,459 | 9,601,969 |
| HF288 | GCCAAT | TGCAT | GI | 13,499,806 | 12,441,532 | 9,475,272 |
| HF289 | GCCAAT | CAACC | GI | 13,501,728 | 12,384,659 | 9,226,314 |
| HF290 | GCCAAT | GGTTG | GI | 12,486,918 | 11,555,725 | 8,684,200 |
| HF291 | GCCAAT | AAGGA | GI | 17,857,208 | 16,349,577 | 12,353,070 |
| HF292 | GCCAAT | AGCTA | GI | 11,618,222 | 10,632,574 | 7,801,685 |
| HF293 | GCCAAT | ACACA | GI | 17,614,268 | 16,153,333 | 12,006,551 |
| HF294 | GCCAAT | AATTA | GI | 12,190,526 | 11,110,161 | 8,170,248 |
| HF295 | GCCAAT | ACGGT | GI | 9,406,598 | 8,676,125 | 6,478,277 |
| HF296 | GCCAAT | ACTGG | GI | 13,877,790 | 12,801,194 | 9,568,606 |
| HF299 | GCCAAT | ACTTC | GI | 8,289,424 | 7,653,972 | 5,705,840 |
| HF453 | GCCAAT | GTAGT | SJI | 10,843,882 | 10,014,890 | 7,495,484 |
| HF454 | GCCAAT | GTCCG | SJI | 5,767,024 | 5,296,414 | 3,931,979 |
| HF455 | GCCAAT | GTCGA | SJI | 2,867,908 | 2,480,044 | 1,753,743 |
| HF457 | GCCAAT | TACCG | SJI | 3,465,520 | 3,139,583 | 2,371,619 |
| HF458 | GCCAAT | TACGT | SJI | 7,196,364 | 6,586,272 | 4,989,721 |
| HF459 | GCCAAT | TAGTA | SJI | 5,015,646 | 4,581,274 | 3,416,914 |
| HF460 | GCCAAT | TATAC | SJI | 3,758,756 | 3,407,469 | 2,549,742 |
| HF461 | GCCAAT | TCACG | SJI | 3,235,804 | 2,910,373 | 2,178,418 |
| HF463 | GCCAAT | TCAGT | SJI | 3,488,478 | 3,188,952 | 2,434,291 |
| HF464 | GCCAAT | TCCGG | SJI | 2,870,566 | 2,261,574 | 1,479,260 |
| HF465 | GCCAAT | TCTGC | SJI | 2,549,802 | 2,326,801 | 1,758,477 |
| HF466 | GCCAAT | TGGAA | SJI | 6,456,506 | 5,784,433 | 4,276,216 |
| HF467 | GCCAAT | TTACC | SJI | 3,630,054 | 3,296,844 | 2,506,188 |
| HF428 | GCCAAT | CTGAT | FSJ | 15,064,818 | 13,900,327 | 10,391,366 |
| HF431 | GCCAAT | CTGCG | FSJ | 12,123,828 | 11,181,258 | 8,379,677 |
| HF433 | GCCAAT | CTGTC | FSJ | 10,458,178 | 9,640,429 | 7,229,054 |
| HF435 | GCCAAT | CTTGG | FSJ | 4,572,150 | 4,146,286 | 3,079,469 |
| HF436 | GCCAAT | GACAC | FSJ | 10,850,970 | 9,914,478 | 7,382,198 |
| HF437 | GCCAAT | GAGAT | FSJ | 10,950,890 | 10,104,977 | 7,703,348 |
| HF438 | GCCAAT | GAGTC | FSJ | 13,946,862 | 12,926,249 | 9,779,690 |
| HF441 | GCCAAT | GCCGT | FSJ | 11,841,888 | 10,871,052 | 8,246,047 |
| HF442 | GCCAAT | GCTGA | FSJ | 7,412,336 | 6,576,082 | 4,747,975 |
| HF448 | GCCAAT | GGATA | FSJ | 4,754,894 | 4,248,536 | 3,060,005 |
| HF450 | GCCAAT | GGCCA | FSJ | 10,845,982 | 9,901,512 | 7,348,970 |
| HF308 | GCCAAT | ATACG | CI | 14,703,640 | 13,564,672 | 10,239,639 |
| HF309 | TGACCA | GCTGA | CI | 3,591,612 | 3,113,163 | 2,279,808 |
| HF311 | TGACCA | GGATA | CI | 15,610,180 | 14,265,558 | 10,754,885 |
| HF312 | TGACCA | GGCCA | CI | 9,009,714 | 8,175,718 | 6,192,009 |
| HF313 | TGACCA | GGCTC | CI | 8,953,782 | 8,209,843 | 6,241,393 |
| HF314 | TGACCA | GTAGT | CI | 23,455,576 | 21,588,410 | 15,792,156 |
| HF315 | TGACCA | GTCCG | CI | 3,087,468 | 2,781,751 | 2,084,957 |
| HF316 | TGACCA | GTCGA | CI | 20,244,424 | 18,597,219 | 14,023,881 |
| HF317 | TGACCA | TACCG | CI | 6,583,238 | 5,950,664 | 4,548,884 |
| HF318 | TGACCA | TACGT | CI | 11,673,630 | 10,646,812 | 8,063,181 |
| HF319 | TGACCA | TAGTA | CI | 14,759,360 | 13,520,948 | 9,772,543 |
| HF320 | TGACCA | TATAC | CI | 6,723,342 | 6,086,107 | 4,605,503 |
| HF322 | TGACCA | TCACG | CI | 7,352,430 | 6,669,964 | 5,020,063 |
| HF323 | TGACCA | TCAGT | CI | 4,284,340 | 3,862,192 | 2,910,414 |
| HF324 | TGACCA | TCCGG | CI | 3,988,970 | 3,317,702 | 2,320,967 |
| HF325 | TGACCA | TCTGC | CI | 6,507,418 | 5,915,323 | 4,448,215 |
| HF326 | TGACCA | TGGAA | CI | 3,755,024 | 3,343,372 | 2,476,619 |
| HF335 | GCCAAT | ATTAC | CI | 15,010,054 | 13,720,249 | 10,242,090 |
| HF336 | GCCAAT | CATAT | CI | 17,566,154 | 16,206,841 | 12,275,571 |
| HF337 | GCCAAT | CGAAT | CI | 15,158,714 | 13,962,586 | 10,575,089 |
| HF338 | GCCAAT | CGGCT | CI | 13,092,224 | 12,071,234 | 9,145,901 |
| HF340 | GCCAAT | CGGTA | CI | 13,081,034 | 11,981,127 | 9,058,987 |
| HF341 | GCCAAT | CGTAC | CI | 14,974,632 | 13,790,995 | 10,334,590 |
| HF345 | GCCAAT | CGTCG | CI | 16,700,816 | 15,449,808 | 11,767,156 |
| HF227 | TGACCA | CATAT | PE | 8,564,796 | 7,719,851 | 5,829,028 |
| HF228 | TGACCA | CGAAT | PE | 5,595,484 | 5,023,052 | 3,627,546 |
| HF229 | TGACCA | CGGCT | PE | 6,265,366 | 5,631,214 | 4,188,244 |
| HF230 | TGACCA | CGGTA | PE | 6,346,068 | 5,723,291 | 4,244,360 |
| HF231 | TGACCA | CGTAC | PE | 6,543,836 | 5,951,749 | 4,436,537 |
| HF232 | TGACCA | CGTCG | PE | 6,078,808 | 5,500,300 | 4,034,373 |
| HF233 | TGACCA | CTGAT | PE | 6,784,860 | 6,152,989 | 4,687,836 |
| HF234 | TGACCA | CTGCG | PE | 8,511,738 | 7,759,592 | 5,797,485 |
| HF235 | TGACCA | CTGTC | PE | 7,191,496 | 6,578,433 | 4,927,660 |
| HF236 | TGACCA | CTTGG | PE | 2,844,414 | 2,507,547 | 1,850,362 |
| HF237 | TGACCA | GACAC | PE | 11,962,092 | 10,872,130 | 8,113,828 |
| HF238 | TGACCA | GAGAT | PE | 2,350,818 | 2,082,991 | 1,515,845 |
| HF239 | TGACCA | GAGTC | PE | 4,728,080 | 4,288,255 | 3,239,258 |
| HF240 | TGACCA | GCCGT | PE | 4,347,366 | 3,873,699 | 2,911,434 |
| HF242 | ACAGTG | GGCTC | PE | 8,693,912 | 7,932,887 | 5,811,848 |
| HF243 | ACAGTG | GTAGT | PE | 11,953,024 | 10,914,015 | 8,037,101 |
| HF244 | ACAGTG | GTCCG | PE | 7,286,542 | 6,630,329 | 4,867,885 |
| HF247 | ACAGTG | GTCGA | PE | 5,717,380 | 5,086,850 | 3,648,825 |
| HF248 | ACAGTG | TACCG | PE | 11,185,020 | 10,150,970 | 7,498,551 |
| HF179 | TGACCA | TCGAT | BT | 6,896,356 | 6,291,805 | 4,693,493 |
| HF180 | TGACCA | TGCAT | BT | 5,158,394 | 4,640,509 | 3,438,663 |
| HF181 | TGACCA | CAACC | BT | 5,625,388 | 4,988,906 | 3,661,847 |
| HF182 | TGACCA | GGTTG | BT | 3,979,922 | 3,595,195 | 2,586,593 |
| HF183 | TGACCA | AAGGA | BT | 5,573,018 | 4,978,185 | 3,698,364 |
| HF196 | TGACCA | ACGGT | BT | 7,654,382 | 6,949,443 | 5,165,238 |
| HF197 | ACAGTG | GAGTC | BT | 9,906,858 | 9,053,935 | 6,673,086 |
| HF198 | ACAGTG | GCCGT | BT | 9,087,080 | 8,193,021 | 5,951,580 |
| HF207 | TGACCA | ACTTC | BT | 9,114,260 | 8,296,623 | 6,098,881 |
| HF208 | TGACCA | ATACG | BT | 7,306,254 | 6,596,273 | 4,847,639 |
| HF210 | TGACCA | ATGAG | BT | 6,846,286 | 6,148,469 | 4,563,235 |
| HF211 | TGACCA | ATTAC | BT | 7,517,370 | 6,780,182 | 5,070,961 |
| HF212 | ACAGTG | GCTGA | BT | 4,299,662 | 3,679,314 | 2,527,040 |
| HF213 | ACAGTG | GGATA | BT | 5,463,162 | 4,904,064 | 3,587,860 |
| HF130 | ATCACG | GTCCG | PuE | 4,661,018 | 4,272,489 | 3,199,431 |
| HF131 | ATCACG | GTCGA | PuE | 4,354,914 | 3,958,142 | 2,945,666 |
| HF132 | ATCACG | TACCG | PuE | 2,760,750 | 2,499,628 | 1,874,672 |
| HF133 | ATCACG | TACGT | PuE | 5,729,468 | 5,232,023 | 3,926,697 |
| HF134 | ATCACG | TAGTA | PuE | 5,397,468 | 4,946,710 | 3,737,881 |
| HF135 | ATCACG | TATAC | PuE | 4,874,586 | 4,456,896 | 3,404,405 |
| HF137 | ATCACG | TCACG | PuE | 2,837,196 | 2,566,728 | 1,921,961 |
| HF140 | ATCACG | TCAGT | PuE | 5,386,056 | 4,944,551 | 3,745,741 |
| HF141 | ATCACG | TCCGG | PuE | 5,014,818 | 4,339,636 | 3,131,097 |
| HF142 | ATCACG | TCTGC | PuE | 4,948,952 | 4,534,712 | 3,479,543 |
| HF143 | ATCACG | TGGAA | PuE | 2,348,668 | 2,105,552 | 1,620,637 |
| HF144 | TGACCA | GCATG | PuE | 10,236,040 | 9,247,460 | 6,621,388 |
| HF145 | TGACCA | AACCA | PuE | 11,306,038 | 10,097,504 | 7,242,473 |
| HF146 | TGACCA | CGATC | PuE | 7,729,418 | 7,050,089 | 5,107,541 |
| HF152 | ACAGTG | CGTAC | PuE | 7,211,794 | 6,542,622 | 4,800,406 |
| HF153 | ACAGTG | CGTCG | PuE | 7,758,604 | 7,017,917 | 5,062,350 |
| HF155 | ACAGTG | CTGAT | PuE | 5,773,146 | 5,191,943 | 3,728,130 |
| HF162 | ACAGTG | CTGCG | PuE | 7,046,958 | 6,364,388 | 4,693,899 |
| HF163 | ACAGTG | CTGTC | PuE | 9,051,986 | 8,267,182 | 6,081,882 |
| HF164 | ACAGTG | CTTGG | PuE | 5,934,996 | 5,343,852 | 3,858,577 |
| HF101 | ATCACG | GAGAT | BA | 5,122,910 | 4,682,490 | 3,537,740 |
| HF102 | ATCACG | GAGTC | BA | 6,931,372 | 6,374,413 | 4,820,276 |
| HF105 | ACAGTG | ACTTC | BA | 15,367,964 | 14,005,176 | 10,105,788 |
| HF106 | ATCACG | GCCGT | BA | 6,496,322 | 5,917,404 | 4,492,540 |
| HF109 | ACAGTG | ATACG | BA | 5,181,180 | 4,626,775 | 3,364,609 |
| HF111 | ATCACG | GCTGA | BA | 5,345,382 | 4,797,135 | 3,595,118 |
| HF120 | ACAGTG | CATAT | BA | 7,622,120 | 6,811,168 | 4,975,059 |
| HF124 | ATCACG | GGATA | BA | 2,173,756 | 1,939,426 | 1,457,610 |
| HF125 | ACAGTG | CGGCT | BA | 7,082,108 | 6,360,942 | 4,625,995 |
| HF126 | ACAGTG | CGGTA | BA | 6,620,480 | 5,939,659 | 4,302,452 |
| HF127 | ATCACG | GGCCA | BA | 7,309,890 | 6,661,305 | 5,046,748 |
| HF128 | ATCACG | GGCTC | BA | 6,788,436 | 6,245,445 | 4,743,598 |
| HF129 | ATCACG | GTAGT | BA | 5,837,214 | 5,355,943 | 4,041,233 |
| HF53 | ATCACG | ATACG | BA | 5,400,962 | 4,918,167 | 3,702,208 |
| HF54 | ATCACG | ATGAG | BA | 5,069,858 | 4,616,998 | 3,452,175 |
| HF56 | ATCACG | ATTAC | BA | 6,528,496 | 5,925,480 | 4,378,424 |
| HF58 | ATCACG | CATAT | BA | 7,977,952 | 7,280,536 | 5,562,587 |
| HF59 | ATCACG | CGAAT | BA | 7,916,800 | 7,232,875 | 5,449,228 |
| HF62 | ATCACG | CGGCT | BA | 1,984,682 | 1,771,942 | 1,314,486 |
| HF63 | ATCACG | CGGTA | BA | 3,426,444 | 3,090,390 | 2,308,728 |
| HF64 | ATCACG | CGTAC | BA | 6,303,500 | 5,756,708 | 4,356,195 |
| HF67 | ACAGTG | GGTTG | BA | 5,388,250 | 4,873,028 | 3,480,196 |
| HF68 | ACAGTG | AAGGA | BA | 9,846,206 | 8,854,048 | 6,495,178 |
| HF69 | ACAGTG | AGCTA | BA | 7,581,318 | 6,811,200 | 5,034,445 |
| HF70 | ACAGTG | ACACA | BA | 5,182,080 | 4,611,159 | 3,384,733 |
| HF71 | ACAGTG | AATTA | BA | 8,032,156 | 7,213,221 | 5,231,500 |
| HF72 | ACAGTG | ACGGT | BA | 7,902,306 | 7,150,612 | 5,278,189 |
| HF74 | ATCACG | CTGAT | BA | 6,622,262 | 6,059,402 | 4,501,857 |
| HF75 | ATCACG | CTGCG | BA | 4,463,390 | 4,076,664 | 3,103,692 |
| HF77 | ATCACG | CTGTC | BA | 3,482,692 | 3,189,118 | 2,394,533 |
| HF79 | ATCACG | CTTGG | BA | 6,459,420 | 5,919,333 | 4,538,405 |
| HF82 | ATCACG | GACAC | BA | 3,511,806 | 3,170,622 | 2,342,801 |
| HF01 | ATCACG | GCATG | LB | 5,129,338 | 4,615,765 | 3,466,597 |
| HF02 | ATCACG | AACCA | LB | 2,422,204 | 2,058,934 | 1,491,924 |
| HF03 | ATCACG | CGATC | LB | 2,112,038 | 1,902,461 | 1,442,324 |
| HF05 | ATCACG | TGCAT | LB | 6,215,944 | 5,670,104 | 4,333,622 |
| HF06 | ATCACG | CAACC | LB | 2,649,432 | 2,354,191 | 1,770,239 |
| HF07 | ATCACG | GGTTG | LB | 2,064,394 | 1,856,080 | 1,375,632 |
| HF08 | ATCACG | AAGGA | LB | 1,241,086 | 1,053,777 | 760,042 |
| HF09 | ATCACG | AGCTA | LB | 3,777,634 | 3,397,857 | 2,584,555 |
| HF11 | ATCACG | ACACA | LB | 6,471,492 | 5,890,962 | 4,451,366 |
| HF12 | ATCACG | AATTA | LB | 5,848,666 | 5,307,905 | 4,029,301 |
| HF13 | ATCACG | ACGGT | LB | 5,480,850 | 4,998,573 | 3,815,291 |
| HF14 | ATCACG | ACTGG | LB | 1,795,384 | 1,595,369 | 1,192,818 |
| HF18 | ACAGTG | GCATG | LB | 4,740,218 | 4,162,444 | 3,005,715 |
| HF19 | ACAGTG | AACCA | LB | 5,493,286 | 4,700,419 | 3,363,842 |
| HF21 | ACAGTG | CGATC | LB | 5,581,312 | 5,046,012 | 3,654,390 |
| HF22 | ACAGTG | TCGAT | LB | 6,493,166 | 5,914,724 | 4,295,552 |
| HF24 | ACAGTG | CAACC | LB | 6,353,446 | 5,636,066 | 4,030,589 |
| HF25 | ATCACG | TCGAT | LB | 2,995,842 | 2,731,670 | 2,108,732 |

**Supplementary Table S3.** Alignment summary statistics.

| **Reference genome alignment** | ***Haliotis fulgens*** | |
| --- | --- | --- |
|  | **Raw Alignment** | **Quality Filters** |
| QC-passed reads | 1,652,799,369 | 1,277,832,122 |
| Secundary | 0 | 0 |
| Supplementary | 26,104,083 | 0 |
| Duplicates | 0 | 0 |
| Mapped | 1,646,591,092 | 1,277,832,122 |
| Paired in sequencing | 1,626,695,286 | 1,277,832,122 |
| Properly paired | 1,514,205,678 | 1,277,832,122 |
| With itself and mate mapped | 1,618,200,970 | 1,277,832,122 |
| Singletons | 2,286,039 | 0 |
| With mate mapped to a different chr | 59,330,550 | 0 |
| With mate mapped to a different chr (mapQ>=5) | 37,588,471 | 0 |

**Supplementary Table S4.** Stacks *ref_map.pl* module summary statistics.

| ***ref_map.pl*** | ***gstacks*** |
| --- | --- |
| **Alignments** | 1,232,450,785 |
| **Primary alignments** | 1,193,407,577 |
| **Soft clipped** | 39,043,208 |
| **Loci** | 464,889 |
| **SNPs** | 1,602,992 |
| **Mean coverage** | 50.4x |
| **Stdev** | 39.2x |
| **Min** | 4.3x |
| **Max** | 184.3x |
| **Mean no. sites/locus** | 255.8 |

**Supplementary Table S5.** Stacks *populations* module summary statistics to final SNP dataset.

| ***populations*** | **SNPS** |
| --- | --- |
| **Total loci** | 464,889 |
| **Removed loci** | 443,132 |
| **Kept loci** | 21,757 |
| **SNPs** | 9,683 |
| **Missing Data** | 9,587 |
| **LD** | 470 |
| **Total SNPs** | 9,117 |

**Supplementary Table S6.** Summary of bioinformatics process and SNPs filtering in comparison with previous research.

| **Modules and Filters** | **Current Study** | **Mejía-Ruíz *et al*., 2020** |
| --- | --- | --- |
| Loci assembles method | *ref_map.pl* | *denovo_map.pl* |
| *Process_radtags:* Phred Score | 25 | 10 |
| Total Sequences | 2,108,074,272 | 1,420,020,148 |
| BWA Alignments | 1,277,832,122 | NA |
| Loci detected | 1,602,992 | 353,332 |
| *Populations:* *p* | 100% | 100% |
| SNP's | 9,117 | 2,216 |
| Missing Data per *loci* | 10% | NA |
| Missing Data per ind | 17% | 10% |
| Neutral markers data det | 9,100 | 2,170 |
| *F_ST_* outliers’ data set | 17 | NA |

**Supplementary Table S7.** Pairwise *F_ST_* for green abalone (*Haliotis fulgens*) with 9,100 neutral loci.

|  | **DPH** | **SCnaI** | **EN** | **GI** | **SJI** | **FSJ** | **CI** | **PE** | **BT** | **PuE** | **BA** | **LB** |
| --- | --- | --- | --- | --- | --- | --- | --- | --- | --- | --- | --- | --- |
| **DPH** | NA | 0.19 | 0.00 | 0.00 | 0.00 | 0.00 | 0.00 | 0.00 | 0.00 | 0.00 | 0.00 | 0.00 |
| **SCnaI** | 0.000 | NA | 0.00 | 0.00 | 0.00 | 0.00 | 0.00 | 0.00 | 0.00 | 0.00 | 0.00 | 0.00 |
| **EN** | 0.005 | 0.007 | NA | 0.00 | 0.00 | 0.00 | 0.00 | 0.00 | 0.00 | 0.00 | 0.00 | 0.00 |
| **GI** | 0.054 | 0.056 | 0.060 | NA | 0.00 | 0.00 | 0.00 | 0.00 | 0.00 | 0.00 | 0.00 | 0.00 |
| **SJI** | 0.038 | 0.040 | 0.044 | 0.024 | NA | 0.00 | 0.00 | 0.00 | 0.00 | 0.00 | 0.00 | 0.00 |
| **FSJ** | 0.050 | 0.053 | 0.056 | 0.023 | 0.007 | NA | 0.00 | 0.00 | 0.00 | 0.00 | 0.00 | 0.00 |
| **CI** | 0.052 | 0.055 | 0.058 | 0.019 | 0.010 | 0.005 | NA | 0.22 | 0.10 | 0.39 | 0.02 | 0.5 |
| **PE** | 0.054 | 0.056 | 0.059 | 0.019 | 0.011 | 0.006 | 0.000 | NA | 0.27 | 0.15 | 0.03 | 0.2 |
| **BT** | 0.055 | 0.057 | 0.060 | 0.021 | 0.012 | 0.006 | 0.001 | 0.001 | NA | 0.53 | 0.21 | 0.3 |
| **PuE** | 0.054 | 0.056 | 0.059 | 0.021 | 0.010 | 0.006 | 0.000 | 0.001 | 0.000 | NA | 0.3 | 0.1 |
| **BA** | 0.053 | 0.056 | 0.059 | 0.020 | 0.011 | 0.004 | 0.001 | 0.001 | 0.000 | 0.000 | NA | 0.4 |
| **LB** | 0.054 | 0.056 | 0.059 | 0.020 | 0.011 | 0.004 | 0.000 | 0.001 | 0.001 | 0.001 | 0.000 | NA |

*F_ST_* values of 9,100 neutral loci are shown below the diagonal and probability values are above the diagonal. Significant values after sequential Bonferroni correction are highlighted in grey (*P* ≤ 0.0036).

**Supplementary Table S8.** Pairwise *F_ST_* for green abalone (*Haliotis fulgens*) with 17 outlier loci.

|  | **DPH** | **SCnaI** | **EN** | **GI** | **SJI** | **FSJ** | **CI** | **PE** | **BT** | **PuE** | **BA** | **LB** |
| --- | --- | --- | --- | --- | --- | --- | --- | --- | --- | --- | --- | --- |
| **DPH** | NA | 1 | 0.18 | 0.00 | 0.00 | 0.00 | 0.00 | 0.00 | 0.00 | 0.00 | 0.00 | 0.00 |
| **SCnaI** | -0.016 | NA | 0.12 | 0.00 | 0.00 | 0.00 | 0.00 | 0.00 | 0.00 | 0.00 | 0.00 | 0.00 |
| **EN** | 0.006 | 0.011 | NA | 0.00 | 0.00 | 0.00 | 0.00 | 0.00 | 0.00 | 0.00 | 0.00 | 0.00 |
| **GI** | 0.351 | 0.357 | 0.302 | NA | 0.00 | 0.00 | 0.00 | 0.00 | 0.00 | 0.00 | 0.00 | 0.00 |
| **SJI** | 0.232 | 0.212 | 0.219 | 0.324 | NA | 0.21 | 0.14 | 0.00 | 0.01 | 0.19 | 0.00 | 0.10 |
| **FSJ** | 0.334 | 0.315 | 0.290 | 0.330 | 0.026 | NA | 0.84 | 0.07 | 0.39 | 0.26 | 0.54 | 0.5 |
| **CI** | 0.366 | 0.348 | 0.337 | 0.376 | 0.030 | -0.012 | NA | 0.00 | 0.44 | 0.62 | 0.88 | 0.6 |
| **PE** | 0.403 | 0.386 | 0.373 | 0.420 | 0.060 | 0.039 | 0.021 | NA | 0.07 | 0.66 | 0.18 | 0.1 |
| **BT** | 0.408 | 0.392 | 0.378 | 0.436 | 0.065 | 0.012 | 0.004 | 0.021 | NA | 0.11 | 0.99 | 0.3 |
| **PuE** | 0.353 | 0.334 | 0.328 | 0.379 | 0.013 | 0.009 | -0.005 | -0.002 | 0.014 | NA | 0.78 | 0.7 |
| **BA** | 0.408 | 0.390 | 0.379 | 0.420 | 0.053 | -0.001 | -0.007 | 0.007 | -0.017 | -0.005 | NA | 0.5 |
| **LB** | 0.372 | 0.357 | 0.343 | 0.372 | 0.036 | -0.001 | -0.003 | 0.013 | 0.006 | -0.010 | -0.001 | NA |

*F_ST_* values of 17 outlier loci are shown below the diagonal and probability values are above the diagonal. Significant values after sequential Bonferroni correction are highlighted in grey (*P* ≤ 0.0019).

**Supplementary Table S9.** Cumulative proportion of explained variance for the environmental Principal Components Analysis (ePCA).

|  | **PC1** | **PC2** | **PC3** | PC4 | PC5 |
| --- | --- | --- | --- | --- | --- |
| Standard deviation | 2.5483 | 1.6565 | 1.08622 | 0.8784 | 2.68891 |
| Proportion of Variance | 0.5411 | 0.2287 | 0.09832 | 0.0643 | 0.03955 |
| Cumulative Proportion | 0.5411 | 0.7698 | **0.86813** | 0.9324 | 0.97197 |

**Supplementary Table S10.** Loadings of the first three PCs retained for the environmental PCA. Selected environmental variables are highlighted in grey shadow.

| **Environmental Variable** | **Abbreviation** | **PC1** | **PC2** | **PC3** |
| --- | --- | --- | --- | --- |
| Mean pH | pH_Mean | -0.2497592 | 0.34176638 | -0.2208187 |
| Min pH | pH_Min | -0.3062434 | 0.21461647 | -0.3461563 |
| Max pH | pH_Max | -0.1960509 | -0.137423 | -0.61094 |
| Mean dissolved oxygen | O2_Mean | -0.3719116 | -0.1443145 | 0.16931869 |
| Min dissolved oxygen | O2_Min | -0.3677287 | -0.0117334 | 0.08597621 |
| Max dissolved oxygen | O2_Max | -0.3442135 | -0.2101287 | 0.16028684 |
| Mean sea surface temperature | SST_Mean | 0.36807635 | 0.19985733 | -0.0328533 |
| Min sea surface temperature | SST_Min | 0.23275352 | 0.41197999 | -0.184847 |
| Max sea surface temperature | SST_Max | 0.33110844 | 0.03338835 | 0.28389187 |
| Mean chlorophyll a | Chla_Mean | 0.2388347 | -0.3924477 | -0.3554521 |
| Min chlorophyll a | Chla_Min | 0.2388347 | -0.3924477 | -0.3554521 |
| Max chlorophyll a | Chla_Max | -0.0016088 | -0.4840624 | 0.16697294 |

**Supplementary Table S11.** Spearman's correlation coefficients among all environmental variables. Positive correlation is shown in blue shadow (≥ 0.5) and negative correlation is shown in red shadow (- ≥ 0.5).

|  | **pH_Mean** | **pH_Min** | **pH_Max** | **O2_Mean** | **O2_Min** | **O2_Max** | **SST_Mean** | **SST_Min** | **SST_Max** | **Chla_Mean** | **Chla_Min** | **Chla_Max** |
| --- | --- | --- | --- | --- | --- | --- | --- | --- | --- | --- | --- | --- |
| **pH_Mean** | 1.00 | 0.87 | 0.26 | 0.45 | 0.65 | 0.27 | -0.39 | 0.14 | -0.46 | -0.61 | -0.61 | -0.28 |
| **pH_Min** | 0.87 | 1.00 | 0.45 | 0.59 | 0.72 | 0.41 | -0.60 | -0.17 | -0.73 | -0.53 | -0.53 | -0.25 |
| **pH_Max** | 0.26 | 0.45 | 1.00 | 0.39 | 0.37 | 0.46 | -0.49 | -0.29 | -0.56 | -0.01 | -0.01 | 0.08 |
| **O2_Mean** | 0.45 | 0.59 | 0.39 | 1.00 | 0.94 | 0.95 | -0.98 | -0.73 | -0.74 | -0.48 | -0.48 | 0.25 |
| **O2_Min** | 0.65 | 0.72 | 0.37 | 0.94 | 1.00 | 0.86 | -0.88 | -0.48 | -0.67 | -0.55 | -0.55 | 0.13 |
| **O2_Max** | 0.27 | 0.41 | 0.46 | 0.95 | 0.86 | 1.00 | -0.95 | -0.75 | -0.71 | -0.38 | -0.38 | 0.24 |
| **SST_Mean** | -0.39 | -0.60 | -0.49 | -0.98 | -0.88 | -0.95 | 1.00 | 0.80 | 0.83 | 0.36 | 0.36 | -0.24 |
| **SST_Min** | 0.14 | -0.17 | -0.29 | -0.73 | -0.48 | -0.75 | 0.80 | 1.00 | 0.58 | 0.02 | 0.02 | -0.49 |
| **SST_Max** | -0.46 | -0.73 | -0.56 | -0.74 | -0.67 | -0.71 | 0.83 | 0.58 | 1.00 | 0.35 | 0.35 | 0.21 |
| **Chla_Mean** | -0.61 | -0.53 | -0.01 | -0.48 | -0.55 | -0.38 | 0.36 | 0.02 | 0.35 | 1.00 | 1.00 | 0.45 |
| **Chla_Min** | -0.61 | -0.53 | -0.01 | -0.48 | -0.55 | -0.38 | 0.36 | 0.02 | 0.35 | 1.00 | 1.00 | 0.45 |
| **Chla_Max** | -0.28 | -0.25 | 0.08 | 0.25 | 0.13 | 0.24 | -0.24 | -0.49 | 0.21 | 0.45 | 0.45 | 1.00 |

**Supplementary Table S12.** Comparative table of GEA marker detection methods for the selected environmental variables.

| **Environmental variables** | **LFMM** | **RDA** | **Consensus** | **In common with *F_ST_* outliers** |
| --- | --- | --- | --- | --- |
| SST_Max | 76 | 30 | 5 | 2 |
| Chla_Ma | 74 | 33 | 7 | 1 |
| pH_Max | 81 | 23 | 7 | 0 |

**Supplementary Table S13.** Assignment accuracies for different SNPs panels and proportion of individuals of *Haliotis fulgens*.

|  |  | **North** | | | **GI** | | | **South** | | |
| --- | --- | --- | --- | --- | --- | --- | --- | --- | --- | --- |
| **panel** | **prop.ind** | **mean** | **median** | **SD** | **mean** | **median** | **SD** | **mean** | **median** | **SD** |
| **50** | 0.70 | 1.00 | 1.00 | 0.00 | 0.24 | 0.20 | 0.20 | 0.98 | 1.00 | 0.03 |
|  | 0.80 | 1.00 | 1.00 | 0.00 | 0.31 | 0.33 | 0.26 | 0.98 | 1.00 | 0.05 |
| **100** | 0.70 | 1.00 | 1.00 | 0.00 | 0.16 | 0.20 | 0.18 | 0.97 | 1.00 | 0.04 |
|  | 0.80 | 1.00 | 1.00 | 0.00 | 0.13 | 0.00 | 0.20 | 0.97 | 1.00 | 0.04 |
| **200** | 0.70 | 1.00 | 1.00 | 0.00 | 0.86 | 0.80 | 0.14 | 1.00 | 1.00 | 0.00 |
|  | 0.80 | 1.00 | 1.00 | 0.00 | 0.90 | 1.00 | 0.19 | 1.00 | 1.00 | 0.00 |
| **300** | 0.70 | 1.00 | 1.00 | 0.00 | 0.83 | 0.80 | 0.18 | 1.00 | 1.00 | 0.00 |
|  | 0.80 | 1.00 | 1.00 | 0.00 | 0.89 | 1.00 | 0.19 | 1.00 | 1.00 | 0.00 |
| **500** | 0.70 | 1.00 | 1.00 | 0.00 | 0.87 | 1.00 | 0.15 | 1.00 | 1.00 | 0.00 |
|  | 0.80 | 1.00 | 1.00 | 0.00 | 0.96 | 1.00 | 0.12 | 0.95 | 1.00 | 0.00 |
| **800** | 0.70 | 1.00 | 1.00 | 0.00 | 1.00 | 1.00 | 0.00 | 1.00 | 1.00 | 0.00 |
|  | 0.80 | 1.00 | 1.00 | 0.00 | 0.98 | 1.00 | 0.07 | 1.00 | 1.00 | 0.00 |
| **1000** | 0.70 | 1.00 | 1.00 | 0.00 | 1.00 | 1.00 | 0.00 | 1.00 | 1.00 | 0.00 |
|  | 0.80 | 1.00 | 1.00 | 0.00 | 1.00 | 1.00 | 0.00 | 1.00 | 1.00 | 0.00 |

**Supplementary Table S14.** Assignment accuracies using 9,117 SNPs for total and half of the individuals of *H. fulgens*.

|  |  | **North** | | | **GI** | | | **South** | | |
| --- | --- | --- | --- | --- | --- | --- | --- | --- | --- | --- |
| **panel** | **prop.ind** | **mean** | **median** | **SD** | **mean** | **median** | **SD** | **mean** | **median** | **SD** |
| **Total** | 0.70 | 1.00 | 1.00 | 0.00 | 1.00 | 1.00 | 0.00 | 1.00 | 1.00 | 0.03 |
|  | 0.80 | 1.00 | 1.00 | 0.00 | 1.00 | 1.00 | 0.00 | 1.00 | 1.00 | 0.00 |
| **Half** | 0.70 | 1.00 | 1.00 | 0.00 | 1.00 | 1.00 | 0.00 | 1.00 | 1.00 | 0.00 |
|  | 0.80 | 1.00 | 1.00 | 0.00 | 1.00 | 1.00 | 0.00 | 1.00 | 1.00 | 0.00 |
